# The RNA-Binding Proteins SRP14 and HMGB3 Control HIV-1 Tat mRNA Processing and Translation During HIV-1 Latency

**DOI:** 10.3389/fgene.2021.680725

**Published:** 2021-06-14

**Authors:** Georges Khoury, Michelle Y. Lee, Sri H. Ramarathinam, James McMahon, Anthony W. Purcell, Secondo Sonza, Sharon R. Lewin, Damian F. J. Purcell

**Affiliations:** ^1^Department of Microbiology and Immunology, Peter Doherty Institute for Infection and Immunity, University of Melbourne, Melbourne, VIC, Australia; ^2^Infection and Immunity Program, Department of Biochemistry and Molecular Biology, Biomedicine Discovery Institute, Monash University, Clayton, VIC, Australia; ^3^Victorian Infectious Diseases Service, The Royal Melbourne Hospital at the Peter Doherty Institute for Infection and Immunity, Melbourne, VIC, Australia; ^4^Department of Infectious Diseases, The University of Melbourne at the Peter Doherty Institute for Infection and Immunity, Melbourne, VIC, Australia; ^5^Department of Infectious Diseases, Alfred Hospital and Monash University, Melbourne, VIC, Australia

**Keywords:** HIV-1, latency, *tat* mRNA, SRP14, HMGB3, mRNA processing, translation

## Abstract

HIV-1 Tat protein is essential for virus production. RNA-binding proteins that facilitate Tat production may be absent or downregulated in resting CD4^+^ T-cells, the main reservoir of latent HIV in people with HIV (PWH) on antiretroviral therapy (ART). In this study, we examined the role of Tat RNA-binding proteins on the expression of Tat and control of latent and productive infection. Affinity purification coupled with mass spectrometry analysis was used to detect binding partners of MS2-tagged *tat* mRNA in a T cell-line model of HIV latency. The effect of knockdown and overexpression of the proteins of interest on Tat transactivation and translation was assessed by luciferase-based reporter assays and infections with a dual color HIV reporter virus. Out of the 243 interactions identified, knockdown of SRP14 (Signal Recognition Particle 14) negatively affected *tat* mRNA processing and translation as well as Tat-mediated transactivation, which led to an increase in latent infection. On the other hand, knockdown of HMGB3 (High Mobility Group Box 3) resulted in an increase in Tat transactivation and translation as well as an increase in productive infection. Footprinting experiments revealed that SRP14 and HMGB3 proteins bind to TIM-TAM, a conserved RNA sequence-structure in *tat* mRNA that functions as a Tat IRES modulator of *tat* mRNA. Overexpression of SRP14 in resting CD4^+^ T-cells from patients on ART was sufficient to reverse HIV-1 latency and induce virus production. The role of SRP14 and HMGB3 proteins in controlling HIV Tat expression during latency will be further assessed as potential drug targets.

## Introduction

The persistence of a reservoir of resting CD4^+^ T-cells harboring silent provirus is the major impediment toward attaining a cure for HIV-1 (Deeks et al., 2016). Despite two decades of intensive investigation, the mechanisms contributing toward establishment and maintenance of latent infection *in vivo* remain incompletely understood (Khoury et al., 2018a). To date, blocks to the initiation of transcription have been the most widely studied and targeted for reversal of latent infection in clinical trials (Ait-Ammar et al., 2020). In these trials, reactivation of virus in the form of cell-associated HIV-1 RNA is detected but decay of the reservoir is almost never induced (Zerbato et al., 2019). Successful production of HIV-1 protein would be required to prime the immune response after reactivation of virus, and results of these trials suggest that additional blocks are present in the latently infected cell that prevent complete processing of RNA or translation to occur. The production of multiply spliced (MS) mRNA is required for successful production of HIV-1 virions, and these transcripts are not always detected after treatment with latency-reversing agents (Pasternak and Berkhout, 2018). We recently found that production of MS RNA is a better indication of virus reactivation *ex vivo* (Zerbato et al., 2021). In addition, a recent study showed that latent infection in blood CD4^+^ T cells from HIV-1 infected individuals on suppressive therapy was due to blocks in the post-initiation stages of transcription, including elongation, RNA-capping and splicing (Yukl et al., 2018).

The HIV-1 regulatory protein, Tat, is essential for successful transcription of the HIV-1 genome and virus production in natural infection (Ott et al., 2011). Whilst transcription initiation is a Tat-independent process, Tat is required for efficient elongation of transcription as the association of the negative factors DSIF (DRB Sensitivity-Inducing Factor) and NELF (Negative ELongation Factor) with the RNA polymerase II causes promoter-proximal pausing (Ping and Rana, 2001). Tat liberates its co-factor, the positive transcription elongation factor-b (P-TEFb), from its sequestration in the inactive 7SK snRNP complex (Krueger et al., 2010; Muniz et al., 2010), to associate with and phosphorylate DSIF and the carboxy-terminus of the stalled RNA polymerase II resulting in release of the transcriptional complex (Ping and Rana, 2001). Notably, Tat is also involved in the other post-transcriptional processes of RNA polyadenylation and splicing (Chiu et al., 2001, 2002; Schapira et al., 2003; Jablonski et al., 2010) where additional blocks to HIV-1 transcription in latent infection have been suggested. There are multiple pieces of evidence supporting the importance of Tat in diverting the integrated provirus away from latent infection. Fluctuations in the levels of Tat protein are a strong indicator of whether a cell will enter latency (Weinberger et al., 2005; Razooky et al., 2015). Nuclear retention of the multiply spliced RNAs that encode Tat or the presence of low levels of Tat protein contribute to maintaining the cells in a latent state (Lassen et al., 2006). Exogenous Tat delivered into latently infected cells inhibited proviral entry into latency, whilst established latency can be reversed by Tat (Donahue et al., 2012). Over time on suppressive therapy, mutations detrimental to Tat function accumulate, contributing to the persistence of latent provirus (Yukl et al., 2009).

We recently characterized the presence of an RNA regulatory element underlying Tat-encoding sequence, which we termed TIM-TAM (Tat IRES modulator of *tat* mRNA) (Khoury et al., 2020). We showed that TIM-TAM is involved in regulating latent infection, as viruses carrying a silent mutation disrupting the secondary structure of TIM-TAM resulted in a restriction in establishment of latency in primary CD4^+^ T-cells. Furthermore, reactivation of latent HIV-1 from the CCL19 primary cell model (Saleh et al., 2011; Spina et al., 2013) infected with virus carrying mutated TIM-TAM was affected after treatment with PMA/PHA (Phorbol Myristate Acetate/PHytohAemagglutinin). The TIM-TAM was also shown to exhibit the properties of an internal ribosome entry site (IRES), RNA structures that facilitate translation initiation in a cap-independent manner.

Proteins required for processing and translation of *tat* mRNAs may be differentially expressed in cells carrying latent provirus and in cells undergoing productive infection. The recent study by Moron-Lopez et al. (2020) showed that human splice factors were differentially expressed between unstimulated and activated cells from ART-suppressed individuals. Activation of the splice acceptor 3 (SA3) site in the HIV-1 genome is required for production of *tat* mRNA, the use of which is tightly regulated by splicing silencers and enhancers (Saliou et al., 2009). In addition to translation initiation factors, auxiliary proteins unique to a particular IRES element may affect IRES function positively or negatively (Lozano and Martínez-Salas, 2015). Several proteins, including HNRNPA1, DDX3, hRIP and HuR (Monette et al., 2009; Rivas-Aravena et al., 2009; Liu et al., 2011) have been reported to impact the function of the HIV-1 5′UTR IRES element.

In this study, we used affinity purification coupled mass spectrometry analysis to identify cellular RNA-binding proteins that interact with *tat* mRNA during productive and latent infection. Using a principal component analysis-based scoring system, a short-list of thirteen proteins were chosen for follow-up investigation. Knockdown (KD) and overexpression assays were used to investigate the roles of these proteins in latent and productive infection of HIV-1 with a dual-fluorescent reporter virus. The effect of KD of these proteins on the various stages of Tat expression—Tat mRNA splicing, Tat translation and Tat transactivation—were explored using luminescence-based reporter systems. The affinity purification approach and downstream validation assay systems allowed identification of Tat-RNA binding proteins that are differentially expressed in resting and activated CD4^+^ T-cells isolated from people living with HIV on ART and are potential druggable targets for modulation of HIV-1 latency.

## Materials and Methods

### RNP Purification by MS2 Selection Affinity

HIV 5′UTRtat1-Tat (NL4-3, nt 455–743; 5777-6044; 8369–8414) and Tat (nt 5,830–6,044; 8369–8414) fragments were amplified and cloned into NheI and EcoRI restriction sites of pcDNA3.1(-)::MS2 plasmid, upstream of 3× MS2 stem-loops (BamHI-KpnI). RNAs were generated by run-off transcription with T7 MEGAscript kit (Promega) using KpnI linearized plasmids. DNA templates were digested with RQ1 RNase-Free DNase (Promega) and RNA were recovered by lithium chloride precipitation then dissolved in MilliQ water. J-Lat cells [clone 6.3, from NIH AIDS Reagent Program, (Jordan et al., 2003)] were expanded in RPMI supplemented with 10% FBS to 10^8^ cells per sample, left untreated or stimulated with TNF-α (20 ng/mL) for 24 h. Activation of virus expression was validated by flow cytometry (GFP^+^ expression) and p24^C*A*^-ELISA as previously described (Khoury et al., 2020). Cells were then collected and lyzed with ice-cold lysis buffer (50 mM Tris-HCl pH 8, 1 mM EDTA, 150 mM NaCl, 0.1% IgePal) supplemented with protease inhibitors (Roche) for 30 min at 4°C. Protein cell extracts were dialyzed before use against buffer D 1× [Hepes KOH pH 7.9 20 mM, KCl 100 mM, glycerol 20%, EDTA 0.2 mM, DTT 0.5 mM) + MgCl2 3 mM + protease inhibitor tablet (Roche)] for 2 h at 4°C, followed by centrifugation for 10 min at 1,700 × g at 4°C. Five hundred pmol of MS2-tagged RNAs into 100 μl buffer D 1× were denatured by 10 min heating at 65°C, followed by slow cooling at room temperature with addition of 7.75 μl of 62.5 mM MgCl_2_ to a final concentration of 4.5 mM MgCl_2_. After 10 min incubation at room temperature, RNAs were incubated with a fivefold molar excess of purified MBP-MS2 fusion protein at 4°C for 20 min. The RNA-MS2:MS2-MBP complexes formed were incubated with amylose beads (200 μl, GE Healthcare) equilibrated in buffer D for 2 h at 4°C. After three washes with 500 μl of buffer D, 1 mg of protein extract supplemented with 5 μM of yeast tRNAs (Sigma-Aldrich) was added. After 20 min of incubation at 4°C with constant agitation, three successive washes were performed in Buffer D and RNP complexes eluted twice by incubation for 20 min at room temperature with 200 μl of Buffer D containing 10 mM maltose. Half of the eluted RNP complexes were processed in solution for mass spectrometry analyses. For western-blot analysis, 10% of the eluted material was used. Experiments were repeated three independent times using different batches of RNA and protein lysate.

### Mass Spectrometry Analysis

TCEP (10 mM, Thermo Fisher Scientific) was added to the eluted sample to reduce the cysteine bonds in proteins, and heated for 20 min at 60°C followed by alkylation of cysteines using 25 mM Iodoacetamide (Sigma-Aldrich) for 20 min at RT in the dark. Samples were subjected to proteolytic cleavage by addition of 1 μg of trypsin protease from porcine pancreas per 100 μg of protein and incubated overnight at 37°C under agitation. Reactions were stopped by addition of formic acid and the resultant peptides were concentrated using C18-packed tips (BondElut, Agilent/Varian). The peptides were subject to online trapping using a PepMap100 trap column at 15 μL/min followed by separation on PepMap 100 C18 nanocolumn (50 cm × 75μm) using a 30-min gradient of Buffer B (80% ACN 0.1% Formic acid) over Buffer A (0.1% Formic acid). The online separated peptides were analyzed using a Q-Exactive plus mass spectrometer (Thermo Fisher Scientific, Bremen, Germany). The survey scans were acquired at 70,000 resolution from 375 to 1,800 m/z, the ion accumulation target was set to 3e6 with maximum injection time of 120 ms. A total of 12 most intense ions (with charge more than 2) were sequentially isolated and fragmented by higher-energy collisional dissociation (HCD) set to 27%, at a resolution of 17,500, target of 1e5 ions and maximum injection time of 120 ms. To identify protein groups, data acquired was converted into mgf and searched against Swissprot human database (version 2016_12) using *ProteinPilot software* (v4.0, SCIEX) with the following search parameters: Iodoacetamide alkylation, trypsin enzyme digestion, instrument-specific settings for TripleTOF 5600 + (MS tolerance 0.05 Da, MS/MS tolerance 0.1 Da, charge state + 2– + 5), biological modification probabilistic features on, thorough ID algorithm, and detected protein threshold 0.05. Mass Spectrometry Interaction Statistics (MiST) (Jäger et al., 2011) analysis was conducted to sort proteins based on specificity, reproducibility and abundance over the three replicates. The mass spectrometry proteomics data have been deposited to the ProteomeXchange Consortium via the PRIDE (Perez-Riverol et al., 2019) partner repository with the dataset identifier PXD025782.

### Generation of Knockdown RFP^+^ Jurkat Cell Lines

Three or four Sherwood UltramiR shRNA viral particles (10^6^–10^7^ TU/mL) against each of the 15 protein targets of interest were obtained from TransOmic Technologies. The pZIP (SFFV) shRNA-mir lentivectors constitutively express the short hairpin RNA (shRNA-mir), puromycin selection marker and red fluorescent protein (RFP) driven by the Spleen Focus Forming Virus (SFFV) promoter. Jurkat cells (2.1×10^5^) were transduced with 100 μL of pooled pseudoviruses for each protein target separately at a MOI of 2.5. Spinoculation was conducted at 1,200 × g for 2 h at 23°C in a flat-bottom 96-well plate. Cells were incubated at 37°C for 72 h then sorted on RFP^+^ expression on an Astrios cell sorter (Beckman Coulter). Bulk populations were then kept in culture under 0.7 μg/mL puromycin selection. Single clones expressing high levels of RFP^+^ cells were selected from the bulk population on the BD FACSAria III. Bulk and single clones were maintained under constant puromycin selection then surviving clones were expanded. Cells were collected for RT-qPCR analysis as well as immunoblotting.

### Cloning of Tat Expression Reporter Constructs

For the Tat/GH1 constructs, HIV-1 sequences were derived from pNL4-3 (M. Martin, NIH, Bethesda, MD, United States), GH1 sequences were amplified from pØhGH (Nichols Institute Diagnostics), LucF from pGL4.13[luc2/SV40] (Promega), and LucR from pGL4.73[hRluc/SV40] (Promega). These segments were assembled by SOE-PCR and cloned into the pcDNA3.1- (Invitrogen) backbone via use of the Xho1 and XbaI sites. The NT5C3 splicing constructs were cloned into the pcDNA3.1- backbone through cleavage of NhoI and NheI where the NT5C3 cellular sequences were amplified from HeLa cell genomic DNA and the other components derived as for the Tat/GH1 constructs.

### DNA Transfection of KD RFP + Cells and Luciferase Analysis

Bulk populations or single clones of the KD RFP^+^ Jurkat cell lines (6×10^4^ cells) were plated into a round-bottom 96-well plate. Media was completely removed and cells were transfected with 1.6 μg reporter constructs using DMRIE-C reagent (1:2.5, Invitrogen) in 62.5 μL Opti-MEM (Gibco). For transactivation assays, 200 ng of Tat WT and 1 μg of Tat hGH reporter construct with 300 ng of both LTR-lucFirefly and lucRenilla. For translation assays, 300 ng of Tat-Cap or IRES LucF reporter constructs were used with 300 ng of lucRenilla for normalization. For splicing assays, 300 ng of Tat^+^ or Tat- reporter constructs with 300 ng of LTR-lucFirefly. Three hours post-transfection, DNA:DMRIE-C mix was removed and media replenished. Twenty-four hours post-transfection, cells were collected and luciferase activity was measured following lysis in 40 μL of 1X passive lysis buffer using a FLUOstar plate reader with the dual-luciferase reporter assay (Promega).

### Virus Production and Transduction

R7GEmTB dual color reporter virus was produced by replacing mCherry into R7GEmC [obtained from NIH AIDS Reagent Program, (Calvanese et al., 2013)] with mtagBFP2 fluorescent protein. SRP14 and PTB cDNA were cloned into pInducer10 lentivector (Meerbrey et al., 2011) by replacing tRFP-shRNA cassette with protein ORF-T2A-mtagBFP2. Viral stocks were generated by transfecting the proviral constructs into HEK 293T cells with Lipofectamine 2000 (Invitrogen) in serum free media (Opti-MEM, Gibco). Supernatants were collected after 72 h, clarified by centrifugation and 0.45 μm filtration to clear cell debris. Particles were concentrated using microcon centrifugal filter device (30K, Merck Millipore) or pelleted by overlaying supernatant on a cushion of 20% (w/v) sucrose in TNE buffer (10 mM Tris-HCl pH 8, 1 mM EDTA, 150 mM NaCl) in Ultra Clear Thinwall tubes and centrifugation at 24,200 rpm for 2 h at 4°C (Beckman SW28/SW41Ti rotor). Viral particle pellets were resuspended into Opti-MEM and stored at –80°C. Virus titres were quantified by measuring p24^C*A*^ levels by capture ELISA as previously described (Khoury et al., 2020) and titration into TZM-bl cells (Sarzotti-Kelsoe et al., 2014). KD RFP^+^ Jurkat cells and CD4^+^ T-cells (10^6^ cells) were infected with 40 and 100 ng R7GEmTB virus (+Env 92HT593.1), respectively. Cells were spinoculated at 1,200 × g for 2 h at 23°C in a 96 well flat-bottom plate and incubated at 37°C for 3 days. Cells were collected, washed and resuspended in 100 μL of 1X PBS then loaded onto cover slips that had been pre-coated with poly-L-Lysine 0.01% (Sigma-Aldrich). Cells were incubated for 1 h on the coverslip then rinsed with PBS 1X and fixed with 2% PFA. Cells were treated with glycine 0.2 M for 10 min at room temperature then rinsed with PBS 1X and H_2_O. Coverslips were inverted on a microscope slide containing ProLong Gold Antifade Mountant (Thermo Fisher Scientific). Images were acquired on LSM710 confocal microscope using *Zeiss Zen software*. J-Lat 10.6, 8.4 and A2 (obtained from the NIH AIDS reagent program) were infected via spinoculation at 1,500 × g for 2 h at 23°C followed by 1 h incubation at 37°C before replenishing the culture with fresh media without or with 5 μg/mL doxycycline. Two days post-infection, cells were washed and stained with Near-IR Live/Dead fixable dead cell staining (Invitrogen). Finally, cells were washed, fixed with 1% formaldehyde and acquired using a Fortessa flow cytometry instrument (BD Bioscience). Analysis was performed using *FlowJo Software, version 10.4.2*.

### Ethics

The studies involving the use of blood samples from HIV negative donors were reviewed and approved by the Human Research and Ethics Committees from the University of Melbourne (15-09VIC-03 and 17-08VIC-01). All HIV-1 seronegative donors were recruited by the Red Cross Blood Bank (Melbourne, Australia) and provided written informed consent for the use of their blood products for the research. The use of blood samples from people living with HIV was approved by the Alfred Hospital (HREC214/15) for the study entitled Large volume peripheral blood mononuclear cells (PBMCs) collection by leukapheresis to define HIV persistence in HIV-infected adults. All participants provided informed consent and the protocol was approved by the local Institutional Review Board.

### Participant Details

PBMCs from people living with HIV on ART with a viral load < 20 copies/mL for ≥3 years were collected by leukapheresis (Alfred Hospital, Melbourne, Australia; Supplementary Table 1) and stored in liquid nitrogen. Resting CD4^+^ T-cells (purity > 95%) were isolated from PBMCs by negative selection using CD4^+^ T-cell isolation kit (Miltenyi Biotec) supplemented with anti-CD69 (clone L78, BD) and anti-HLA-DR (clone 2-O6). CD4^+^ T-cells were activated with anti-CD3/CD28 (coated α-CD3 clone OKT3, BD, 1 μg/mL and soluble α-CD28 clone L293, BD, 0.5 μg/mL) for 48 h at 37°C.

### Western-Blot

Resting and activated CD4^+^ T-cells isolated from patients under ART (30×10^6^ cells) were lysed for 30 min on ice using ice-cold IGEPAL cell lysis buffer (Tris-HCl pH 8.8 50 mM, NaCl 150 mM, IGEPAL 1%, EDTA 1 mM) supplemented with protease inhibitor cocktail (Complete^TM^, Mini, EDTA-free Protease Inhibitor Cocktail, Roche). Cell lysate was cleared and quantified using the Bio-Rad Protein Assay Reagent (Bio-Rad). Equal amounts of each sample (20 μg) were loaded on 15% SDS-PAGE, transferred to nitrocellulose membrane. Blots were probed with anti-SRP14 (B-3, sc-377012, Santa Cruz) at 1:10, anti-HMGB3 (clone 546519, R&D) 1:1,000, anti-PTB (clone 7, #325000, Invitrogen) at 1:500 and anti-GAPDH (14C10, Cell Signaling) at 1:1,000. Antibodies were detected using either goat anti-rabbit (Invitrogen, #656120) or goat anti-mouse IgG (H + L) HRP (Invitrogen, #626520) at 1/5,000 and developed with SuperSignal West Pico Chemiluminescent Substrate (Thermo Fisher Scientifc). Images were visualized using an *MF-ChemiBis 3.2 imaging system (DNR)*.

### Quantitative Real-Time PCR Analysis

RNA from resting and activated CD4^+^ T-cells isolated from patients under ART (15×10^6^ cells) and bulk and individual clone of interest for the KD RFP + Jurkat cells as well as untransduced Jurkats were extracted using TRIzol following the manufacturer’s protocol. RNA (500 ng) was DNase-treated with RQ1 DNase (Promega) and reverse transcribed using 4 U OmniScript (Qiagen) with dNTP (0.5 mM), random hexamers (2 μM), oligo(dT)_15_ (1 μM), and RNasin (10 U) in 30 μL reactions for 1 h at 37°C. Real-time PCR was performed on CFX Connect real-time PCR detection system (Bio-Rad) using Fast SYBR Green Master Mix (Applied Biosystems), forward/reverse primers (Supplementary Table 2) at 500 nM final concentration, 2 μL of undiluted cDNA in reactions with a final volume of 20 μL. Each sample was assayed in duplicate and normalized on GAPDH content and untransduced Jurkat cells for KD RFP + Jurkats or 18S content and activated CD4^+^ T-cells for primary CD4^+^ T-cells. Cycling conditions used were 95°C for 10 min for activation of the DNA polymerase followed by 45 cycles of denaturation at 95°C for 3 s and annealing/extension at 60°C for 20 s. Melt curve analysis was performed each time with a starting temperature of 60°C, increasing to 95°C with 0.5°C increments every 0.05 s. Data were analyzed on *CFX Manager 3.0* software employing 2^−ΔΔCt^ to determine fold changes.

### T-Cell Electroporation

Electroporation of resting CD4^+^ T-cells was performed using an Amaxa human T-cell Nucleofector kit (Lonza). Purified resting CD4^+^ T-cells (5.3×10^6^) were re-suspended in 100 μl nucleofector solution (4.5:1 ratio of human T-cell nucleofector solution:supplement) and transfected with 1.2 μg DNA per 10^6^ cell using Amaxa nucleofector program U-014 for high viability. Cells were then transferred into a 12-well plate containing pre-equilibrated media, followed by half-media change 6 h post-nucleofection. Cells were maintained for 48 h at 37°C in media supplemented with 1% FBS and 1 U/mL IL-2, with or without 5 μg/mL doxycycline. After 2 days, cells were collected and stained for CD25 (PE-Cy7, clone 2A3, BD) and HLA-DR (V605, clone L243 BioLegend) activation markers in comparison to PHA stimulated (10 μg/mL) cells. Culture supernatant was also harvested and viral RNA was isolated using QIAamp Viral RNA Mini Kit (Qiagen) following the manufacturer’s protocol. vRNA was DNase treated (2 U/μg RNA, Promega) and reversed transcribed using Omniscript as described above. HIV RNA levels were assessed using droplet digital PCR (ddPCR) using *pol* primers/probe (Supplementary Table 2). Thermal cycling was conducted as follows: 95°C for 10 min, 40 cycles of 94°C for 30 s and 60°C for 60 s, followed by 98°C for 10 min (ramp rate 2°C/s for each step) on a C1000 Touch Thermal cycler (Bio-Rad). The droplets were subsequently read on a QX200 droplet reader (Bio-Rad) and the data were analyzed with *Quanta-Soft 1.7.4 software*. The limit of detection of our assay was of 58 copies/mL.

### Recombinant Proteins

Production and purification of MBP-MS2 protein was done as previously described (Deckert et al., 2006). cDNA encoding SRP14, HMGB3 and PTB were cloned into NheI and HindIII restriction sites of pET28a + bacterial vector (Novagen) allowing the expression of N-terminally 6×-His tagged proteins. All recombinant proteins were expressed in E. coli BL21 (DE3) Codon + bacteria grown in LB/Kanamycin and induced by addition of 0.1 mM IPTG overnight at 37°C (for PTB and SRP14) or 30°C (for HMGB3). Cells were sonicated in lysis buffer containing 20 mM potassium phosphate pH 7.4 (pH to 6 for HMGB3), 1M KCl, 1 mM DTT, 20 mM imidazole and protease inhibitor cocktail (Roche), then treated with 1.5 mg/mL lysozyme (from chicken egg white, Sigma-Aldrich) for 20 min on ice. Soluble proteins were purified by polyethyleneimine (0.4% final) followed by ammonium sulfate precipitation (30% for SRP14 and PTB; 40% then 60% precipitation for HMGB3). Proteins were purified by affinity purification using Ni-NTA affinity resin (Qiagen) coupled to an Äkta pure FPLC (GE Healthcare). The bound proteins were eluted with a linear imidazole gradient (20 mM to 500 mM). Proteins were buffer exchanged into buffer D (20 mM HEPES–KOH pH 7.9, 100 mM KCl, 0.2 mM EDTA, 3 mM MgCl_2_, 0.5 mM DTT, 20% glycerol) using PD-10 desalting columns (GE Healthcare) followed by concentration using 3, 10, and 30 k cutoff amicon columns (Merck) for SRP14, HMGB3 and PTB, respectively. Proteins concentration was determined using UV spectroscopy at 280 nm. Purity was confirmed by 12.5% SDS-PAGE and western-blot analysis using protein specific antibodies and anti-His HRP conjugated antibody (clone J099B12, BioLegend) at 1:500.

### Footprinting Assays

Footprinting analysis was performed by SHAPE as previously described (Mortimer and Weeks, 2007). Briefly, *tat2* RNA was generated by run-off transcription using Sp6 MEGAscript kit (Promega) and pSP65::tat2 construct linearized with ClaI. *In vitro* transcribed *tat2* RNA (1 pmol) was probed in 3× folding buffer (333 mM HEPES–KOH pH 8, 333 mM NaCl, 33.3 mM MgCl_2_) in the presence or absence of SRP14, HMGB3 or PTB recombinant protein at 5, 10 and 20 protein/RNA molar ratio (0.41–1.6 μM). RNP complexes were formed by incubation for 20 min at room temperature then probed with 1-methyl-7-nitroisatoic anhydride (1M7, 65 mM in DMSO) or DMSO for 4 min at 37°C, then recovered by ethanol precipitation. Primer extension was conducted as described previously with 0.4 μM fluorescently labeled odp3102 primer (6-FAM or HEX, Sigma-Aldrich, Supplementary Table 2). The dideoxy sequencing reactions were generated using unmodified RNA, labeled primers (PET or NED, Applied Biosystems) and 0.5 mM ddGTP. cDNAs were recovered by ethanol precipitation and separated by capillary electrophoresis with LIZ500 size standard (ABI 3130, AGRF). Data was processed using the *QuShape software* (Karabiber et al., 2013). Protections induced by SRP14, HMGB3 or PTB binding were indicated by a reduction in the normalized SHAPE reactivities.

## Results

### Detection of 243 Putative Tat-RNA and Cellular Protein Interactions

**FIGURE 1:**
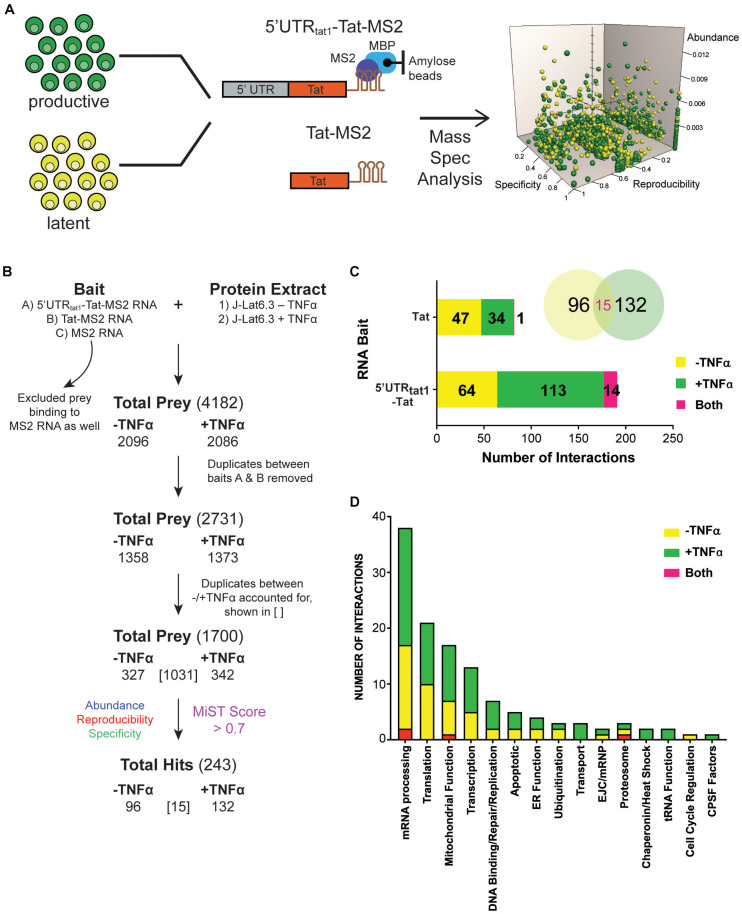
Enrichment in mRNA processing and translation factors assembling on *tat* mRNA. **(A)** Representation of the affinity purification strategy used for isolation of cellular RNA binding proteins that interact with *tat* mRNA. Whole cell lysates were prepared from the latently infected T-cells, J-Lat clone 6.3 where untreated cells were used as the latent sample and TNF-α activated cells were used as the productively infected sample. Cellular proteins that interact with *tat* mRNA were pulled down through MBP-MS2 affinity purification using two RNA baits that contained Tat exon 2 with or without the 5′UTR from tat1 mRNA and fused to three MS2 sequences. Eluted RNA-protein complexes were analyzed by mass spectrometry. MiST analysis was used to determine the most biologically relevant interactions after combining data from three replicates. **(B)** The filtering steps applied to refine the mass spectrometry data. Prey binding to the control bait, MS2 RNA were excluded, followed by removal of duplicate prey captured by baits A (5′UTRtat-Tat) and B (Tat RNA). Prey were then defined as derived from uniquely –TNFa or +TNFa lysates or from both. Lastly, MiST analysis was applied to score interactions and a confidence threshold of 0.7 was used to refine the list of candidates for relevant Tat mRNA: cellular protein interactions. **(C)** Breakdown of the 243 proteins selected by MiST analysis by bait and lysate. **(D)** Annotation of the 243 proteins based on GO biological process.

To explore MS RNA binding partners during latency, we initiated a proteomic approach based on affinity chromatography purification of RNA-protein complexes (Maenner et al., 2010; Bar et al., 2011) formed upon incubation of *in vitro* transcribed *tat* RNA with protein lysate, followed by protein identification by mass spectrometry. An overview of the processes is shown in Figure 1A. Protein lysates were prepared from the HIV-1 latently infected T cell line, J-Lat6.3 where the cells were either left untreated (latent infection) or activated with TNF-α (productive infection). The RNA of interest, 5′UTRtat1-Tat and Tat with three binding sites for the MS2 coat protein fused at their 3′-ends were used as baits. In parallel, a control RNA that only contained the three MS2 binding sites was used. RNA retention to amylose beads was mediated by the MS2-MBP fusion protein containing the MS2 coat protein RNA binding domain and the *E. coli* maltose binding protein (MBP). RNP complexes were eluted by maltose and the protein composition of the purified RNPs were analyzed by mass spectrometry. The proteins identified by MS were quantitatively scored using the mass spectrometry interaction statistics (MiST) platform devised by Jäger et al. (2011).

Several filtering steps described in Figure 1B were applied to refine the protein preys obtained by MBP-MS2 pull-down for generation of a list of 1,700 unique proteins where duplicates between the different conditions were accounted for. These 1,700 proteins were quantitatively scored based on their abundance, reproducibility and specificity and 243 putative Tat RNA:protein interactions were identified using a confidence threshold of 0.7 for biological relevance (Figure 1B).

As expected, when assigned to their respective bait (Figure 1C, bar graph) or protein lysate (Figure 1C, Venn Diagram), we observed a larger number of proteins interacting with the longer RNA bait (191 proteins interacting with the 5′UTRtat1-Tat-MS2 vs. 82 with Tat-MS2). Moreover, a higher number of interactions were detected with the lysates prepared from activated T-cells (147 proteins for +TFNα vs. 111 proteins for −TNFα). Analysis of the 243 proteins by broad gene ontology terms (Spliceosome database, Cvitkovic and Jurica, 2013) showed that the top two overrepresented annotated biological processes were mRNA processing and translation (Figure 1D). Analysis of the GO molecular function also showed a predominance of RNA-binding proteins (Supplementary Figure 1).

### Knockdown of Tat RNA Binding Proteins Affect Latent and Productive Infection of HIV-1

**FIGURE 2:**
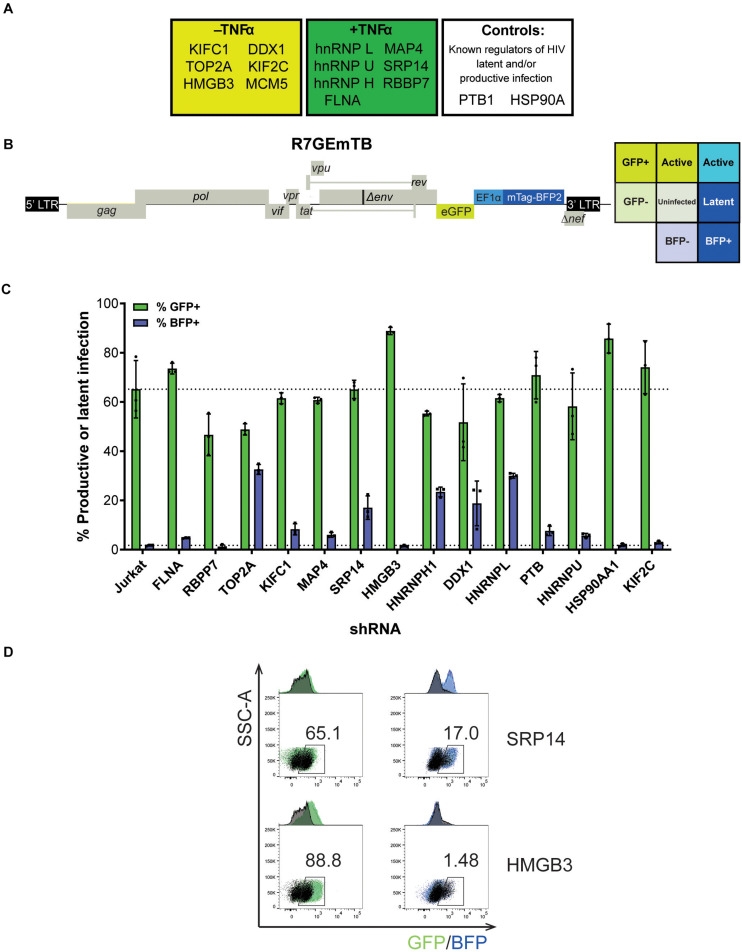
SRP14 and HMGB3 knockdown impact HIV latent and productive infection. **(A)** Short-list of 13 proteins chosen for follow-up validation and two control proteins, PTB and HSP90A that are known regulators of HIV-1 replication. **(B)** Genome of the single round DuoFluo virus [R7/E-/GFP-EF1α-mTagBFP2 (R7GEmTB)] used in this study. The fluorescence profiles that correspond to latent or productive infection are shown in the grid. **(C)** Bulk populations of RFP^+^ shRNA knockdown (KD) Jurkats were infected with the single round DuoFluo virus pseudotyped with the dual-tropic 92HT593.1 envelope, and collected 48 h later for flow cytometry analysis. Percentage of productive or latently infected cells after knockdown of the specific protein is shown, with productive infection in green and latent infection in blue. Data shown are means of three independent experiments ± SEM. **(D)** Scatter plots highlighting the shift in GFP^+^ or BFP^+^ populations after infection of RFP^+^ SRP14 (top) or HMGB3 (bottom) KD Jurkats with DuoFluo virus. Black population represent infected, untransduced Jurkat cells, while green/blue populations represent infected SRP14/HMGB3 KD RFP^+^ Jurkats. Values represent the percentage of GFP^+^ or BFP^+^ cells in KD RFP^+^ Jurkat cells.

From the 243 proteins identified through MS2 chromatography affinity purification, thirteen proteins were selected to follow through with validation studies (Figure 2A and Supplementary Table 3). The main criteria for selection were the MiST score, the role of the protein in cellular pathways and the novelty of the protein in the context of regulation of HIV-1 latent infection. Two additional proteins, PTB1 and HSP90A were selected as controls as both proteins have previously been shown to be involved in regulation of HIV-1 latency (Lassen et al., 2006; Anderson et al., 2014).

For further refinement of the list of candidates for in-depth investigation, we assessed the effect of knockdown of the proteins on HIV-1 latent and productive infection. Jurkat T-cells were transduced with shRNA expressing lentivectors targeting each of the fifteen protein targets for knockdown (KD). MCM5 KD induced high cell death hence MCM5 was excluded from further investigation. Successfully transduced cells were sorted into a bulk population by RFP^+^ expression (Supplementary Figure 2A) where the degree of knockdown was heterogeneous between individual cells. Success of the knockdown of the gene targets in the bulk Jurkat cell populations were confirmed by western blot and densitometry analysis (Supplementary Figure 2B). To assess the outcome of protein knockdown on HIV-1 infection, we used a single-round dual-fluorescent reporter virus (DuoFluo, R7GEmTB) based on the R7GEmC backbone described in Calvanese et al. (2013). In our DuoFluo virus, HIV-1 5′-LTR controls eGFP expression indicative of productive infection and mTagBFP2, controlled by the EF1α promotor, is the marker of latent infection (Figure 2B). The fluorescent phenotype of cells and corresponding profile of HIV-1 infection are shown in the grid (Figure 2B). We confirmed the ability of the eGFP/mTagBFP2 expressing dual-fluorescent reporter virus to identify the presence of latently (blue, BFP^+^) and productively (green, GFP^+^ or cyan, GFP^+^ BFP^+^) infected Jurkat and primary CD4^+^ T-cells by fluorescence microscopy after infection with R7GEmTB (Supplementary Figure 3).

All bulk populations of the KD RFP^+^ Jurkat cells were infected with the DuoFluo virus and collected for flow cytometry analysis after 72 h. Levels of eGFP and mTagBFP2 expression were assessed by gating on the RFP^+^ population and compared against DuoFluo infected untransduced RFP- parental Jurkat cells. An increase in productive infection was detected after knockdown of FLNA, HMGB3, PTBP1, HSP90AA1, and KIF2C (Figure 2C, green bars). On the other hand, a dramatic increase in latent infection was seen after knockdown of TOP2A, SRP14, HNRNPH1, DDX1, and HNRNPL (Figure 2C, blue bars). The increase in latent infection after knockdown of TOP2A and HNRNPH1 was coupled with a decrease in productive infection.

PTB, known to facilitate the export of multiply spliced (MS) mRNAs to the cytoplasm (Lassen et al., 2006), appears to play a role in controlling latent and productive infection, as increases in both forms of infection were observed after knockdown of the protein (productive: 70.9%, latent: 7.61%). However, the effect on latent infection was smaller than expected. Interestingly, two genes, SRP14 and HMGB3 have not been previously reported to play a role in the regulation of HIV-1 replication and their knockdown here had very marked effects on latent and productive infection, respectively. Knockdown of SRP14 had no effect on productive infection but increased dramatically the percentage of cells entering into latency (17.0% vs. Jurkat 1.71%, Figure 2D). In contrast, knockdown of HMGB3 had no effect on latent infection, but increased the percentage of productively infected cells (88.8% vs. Jurkat 65.2%, Figure 2D). These data suggest that SRP14 is a negative regulator of latent infection, whilst HMGB3 is a negative regulator of productive infection.

### Knockdown of SRP14 and HMGB3 Strongly Modulates Splicing at SA3

**FIGURE 3:**
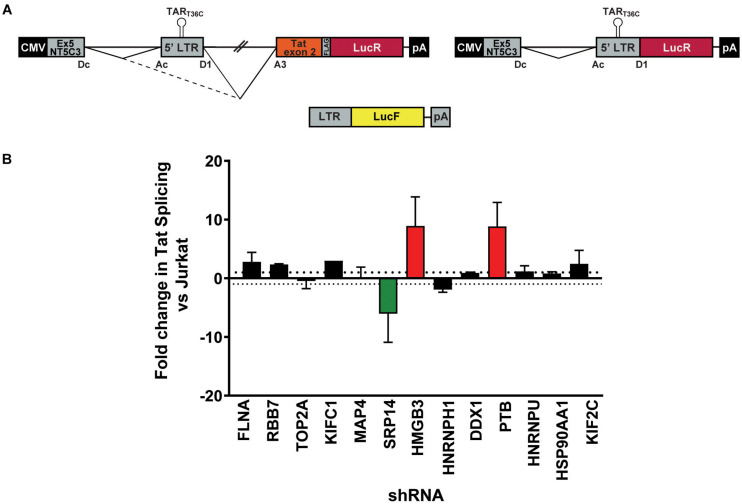
SRP14 and HMGB3 knockdown influence *tat* mRNA splicing. **(A)** Schematic representation of the NT5C3-Tat splicing reporter construct (Tat+), the corresponding Tat- control and the transactivation reporter cassette (LTR-LucF). The splicing patterns that lead to SA3 activation and Tat expression are indicated. RFP^+^ shRNA KD Jurkats were co-transfected with either the Tat^+^ or Tat– splicing reporter vectors and LTR-LucF, and 24 h later harvested for luciferase readout. **(B)** The fold changes in Tat splicing (Luciferase Firefly/Renilla ratio for Tat^+^ vs. Tat– cells) over untransduced Jurkat cells were determined. Data represent mean of three independent experiments ± SEM. D, donor; A, acceptor splice site.

As a strong block to multiply splicing of HIV-1 mRNA was recently characterized in CD4^+^ T-cells isolated from patients under ART (Yukl et al., 2018), we examined the role of knockdown of the RNA binding proteins on *tat* mRNA splicing using an HIV-1 splicing reporter, which harbors SD1 5′ss and SA3, SA4a,b,c and SA5 3′ss (Ropers et al., 2004). In this splicing reporter construct, Tat-exon1,2 (nt 1-839/5590-6044 NL4-3) was placed in the context of a human gene to recapitulate HIV integration in latently infected cells. The context of HIV-1 integration in the latent cell line, ACH2 cells (Clouse et al., 1989, Folks et al., 1989) was used as the basis of the design hence NT5C3 exon 5 and intron 5 were incorporated upstream of the HIV-1 5′-LTR (Figure 3A). Renilla luciferase (LucR) was introduced at the 3′ end of Tat exon 2, thus an increase in LucR would indicate splicing at SA3, SA4a,b,c or SA5. Co-transfection of the splicing reporter with an LTR-LucF reporter cassette allows Firefly luciferase to be used as a specific readout of splicing at SA3 and Tat production. The effect of cellular proteins on use of SA3 in the Tat^+^ splicing reporter were compared to a matched Tat- splicing reporter lacking both the HIV introns and Tat exon 2 (Figure 3A). An increase in LucR expression in the Tat- context shows the promotion of splicing between the cellular splice site, SDc site at the 3′ end of NT5C3 exon 5 and the SAc upstream of the 5′-LTR.

Bulk populations of the KD RFP + Jurkat cells were co-transfected with the Tat^+^ or Tat- splicing reporter and the LTR-LucF cassette and harvested for luciferase analysis 24 h later. The LucF/LucR ratio was calculated for both the Tat^+^ and Tat- contexts in response to the knockdown of each protein target. Fold changes in Tat splicing over transfected RFP- untransduced Jurkat cells were reported in Figure 3B. Knockdown of SRP14 decreased splicing at SA3 by sixfold compared to untransduced Jurkats, whereas knockdown of HMGB3 and PTB increased the use of SA3 by 8.9- and 8.8-fold, respectively (Figure 3B). None of the other ten proteins of interest affected splicing at SA3. The effect of SRP14 and HMGB3 KD on use of SA3 is consistent with the effects of these proteins on latent and productive infection, suggesting an important role of these proteins in Tat mRNA processing and regulation of HIV-1 infection.

### Knockdown of SRP14 and HMGB3 Impacts Tat Expression and Function

Major blocks of HIV transcription and translation have been reported during latency (Khoury et al., 2018a). Due to the central role of Tat protein in promoting HIV transcription and post-transcriptional events, this warranted a deeper investigation of the role of SRP14, HMGB3 and PTB on Tat expression. One caveat of using bulk populations of the KD RFP^+^ Jurkat cells is the large clonal variation. To circumvent this obstacle, single clones were sorted following 12 days of puromycin selection and assessed through expression of RFP by flow cytometry (Supplementary Figure 2C). To examine KD efficiency in the various clones, changes in mRNA levels compared to untreated Jurkat T-cells was determined by RT-qPCR. Importantly, we observed across all single clones tested a significant reduction in the levels of *SRP14*, *HMGB3* and *PTB* mRNA compared to untransduced Jurkats (FC vs. Jurkat ≥50%, Supplementary Figure 2D).

**FIGURE 4:**
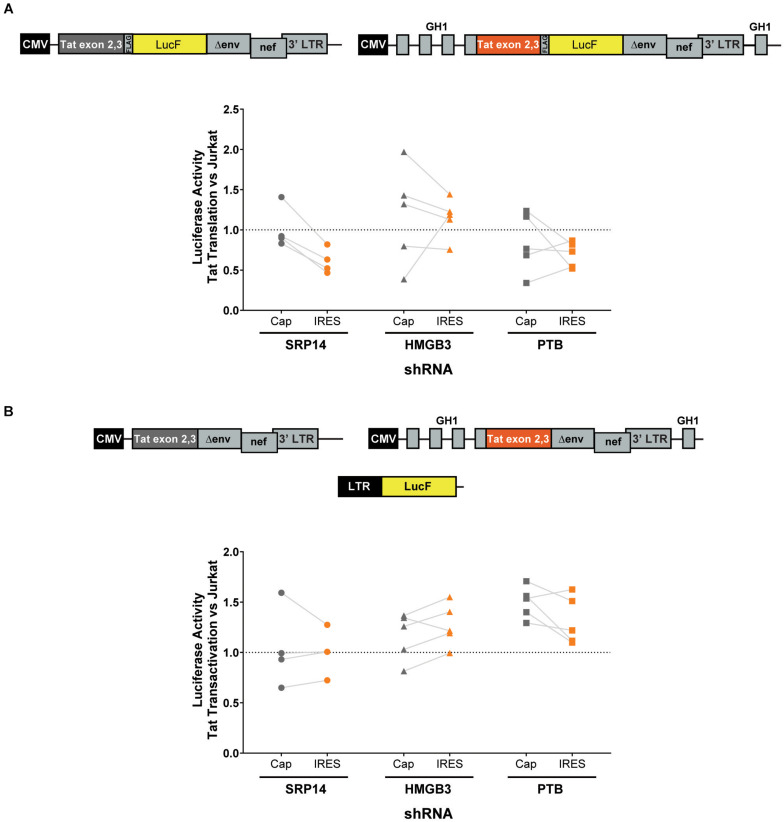
SRP14 and HMGB3 knockdown control Tat -cap and -IRES dependent translation. Diagrams depicting Tat reporter constructs used to study Tat cap- (left) or IRES- (right) dependent translation **(A)** and transactivation **(B)**. Luciferase Firefly expression, which is produced in fusion with Tat (in A) or under the control of the 5′-LTR (in B), is used as a readout for Tat translation and transactivation, respectively. Bulk populations or single clones selected for knockdown of SRP14, HMGB3 or PTBP1 were transfected with Tat cap or IRES expression constructs to assess the effect on Tat translation **(A)** or transactivation **(B)**. Cells were harvested for luciferase analysis 24 h later. Firefly luciferase activity (LucF) was normalized on Renilla luciferase activity (LucR) and shown as a fold change over untransduced Jurkats. Data shows mean of two independent experiments with single clones and bulk populations. CMV, cytomegalovirus promoter; GH1, human growth hormone gene.

Next, bulk and single SRP14 (B5, C10, and G4), HMGB3 (C2, D4, E7, and G9) or PTB (C10, C11, D5, and D8) shRNA KD clones were transfected with Tat expression constructs to assess the effect on Tat transactivation and translation. We previously characterized a highly conserved element underlying the Tat open reading frame, named TIM-TAM (for Tat IRES modulator of *tat* mRNA) and characterized its role in controlling Tat translation through cap- and IRES-dependent mechanisms (Khoury et al., 2020). Moreover, we developed a model system to assess Tat cap and IRES translation (Nguyen et al., 2019). In our IRES-dependent Tat translation expression cassette, the Tat encoding exons have been incorporated into the human growth hormone gene (GH1), where readthrough transcription and alternative splicing would allow low level expression of GH1-Tat protein through an IRES-dependent mechanism (Figure 4A). In the control construct, Tat was placed under the control of a cytomegalovirus (CMV) promoter allowing Tat expression in a cap-dependent manner. In both contexts, Tat was cloned in phase with LucF hence lucF expression is a marker of Tat translation. Bulk population and single clones of the KD RFP^+^ Jurkat cells were transfected with cap or IRES Tat-LucF expression constructs and harvested for luciferase analysis 24 h later. Fold changes in luciferase activity were then calculated in comparison to RFP- untransduced Jurkats. In the SRP14 KD RFP + Jurkats, Tat translation was reduced from the cap-dependent context in the bulk population and 2 of 3 single clones and from the IRES-dependent context in the bulk population and all three single clones (Figure 4A). In contrast, HMGB3 knockdown induced an increase in Tat translation from the cap-dependent context for the bulk population and 2 of 4 single clones, and in the bulk population and 3 of 4 single clones for the IRES-dependent context (Figure 4A). Lastly, knockdown of PTB resulted in a decrease in Tat translation in 2 of 4 single clones transfected with Tat-lucF construct, as well as all 4 single clones and the bulk population transfected with Tat IRES construct (Figure 4A).

We next investigated the effect of SRP14 and HMGB3 KD on Tat transactivation by using a modified expression cassette system, where Tat is translated through an IRES- or cap-dependent pathway and the luminescence readout is induced by Tat transactivation of an HIV-1 LTR-LucF reporter cassette. The effect on Tat transactivation after knockdown of SRP14 was variable across the single clones for both cap- and IRES-Tat translation, however, there was a clear increase in Tat transactivation for 3 out of 4 Tat-cap transfected single clones and 4 out of 4 Tat-IRES transfected single clones after knockdown of HMGB3 compared to untransduced Jurkats (Figure 4B). Knockdown of PTBP1 resulted in an increase in Tat transactivation for all single clones transfected with Tat-Cap and Tat-IRES (Figure 4B). These data demonstrate a role of SRp14 and HMGB3 in controlling HIV-1 latent and productive infection in Tat-dependent manner.

### SRP14 and HMGB3 Binds in the Vicinity of Tat Start Codon

**FIGURE 5:**
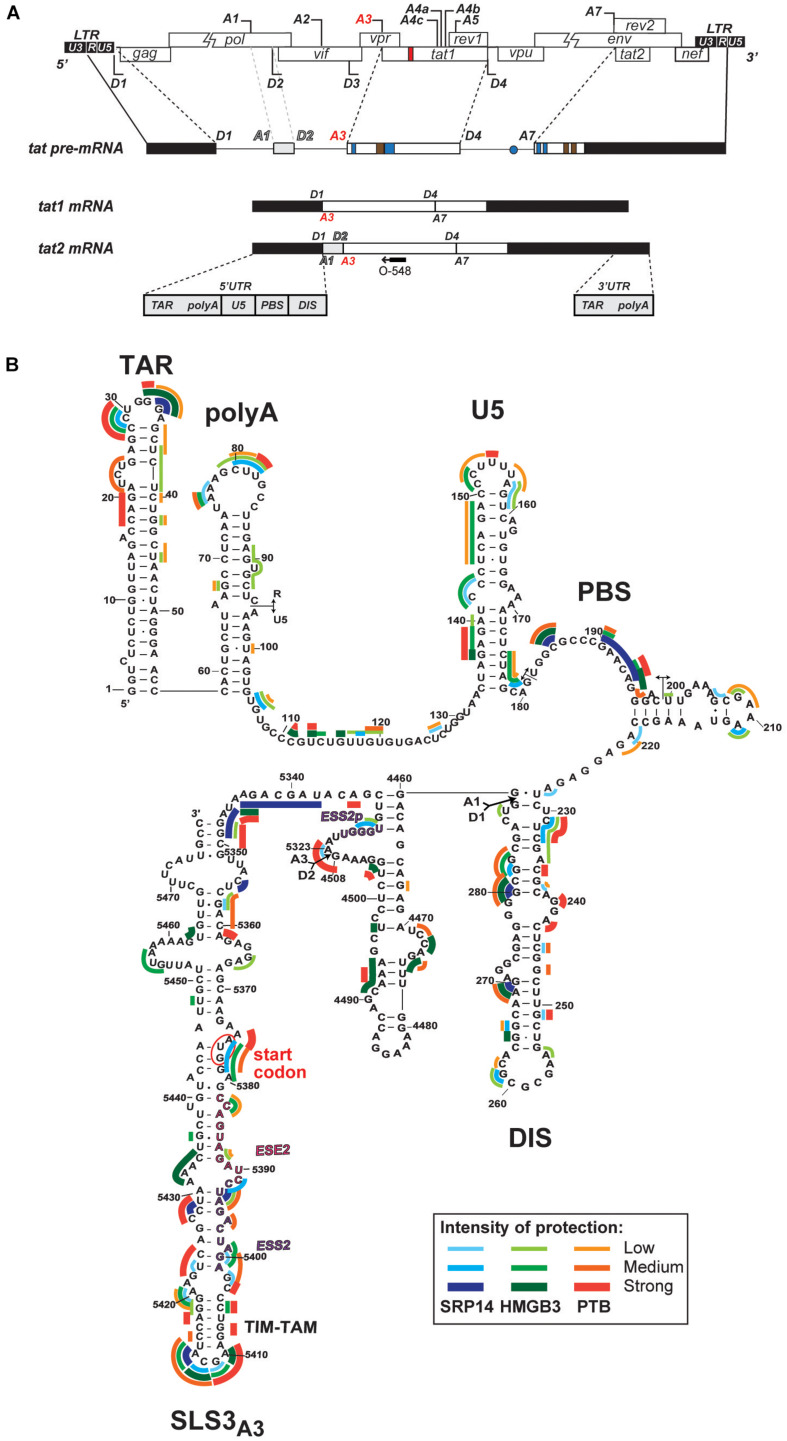
Interaction of SRP14, HMGB3 and PTB with *tat* mRNA. **(A)** Schematic representation of HIV-1 proviral genome, *tat* pre-mRNA and multiple splice events leading to *tat1* and *tat2* mRNAs production during the early phases of HIV-1 infection. D represent donor splice sites, A represent acceptor splice sites, 5′UTR/3′UTR indicate 5′/3′ untranslated regions. The hybridisation site used for primer extension with O-548 (also known as Odp3102) is indicated by black arrow. **(B)** Probing of SRP14, HMGB3, and PTB binding sites on *tat2* mRNA. *Tat2* transcript (1 pmol) was modified with 1M7 (65 mM) in the absence or presence of increasing concentration (0.41—0.83—1.6 μM) of recombinant proteins. Conditions of modification are given in Materials and Methods. Protections generated by SRP14, HMBG3 and PTB recombinant proteins are indicated on the secondary structure model of *tat2* mRNA by blue, green and red lines, respectively. Pale, medium, and dark colors indicate the intensity of protections (low, medium and strong protections). Numbering of nucleotides and positions of the *cis* regulatory elements are given in reference to HIV-1 BRU (K02013). The start codon of Tat protein is circled.

Whilst SRP14 and HMGB3 were detected in the RNP complexes (Supplementary Figure 4), further investigation was required to assess direct interaction of these proteins with *tat* mRNA. To delineate SRP14, HMGB3, and PTB binding sites on multiply spliced RNA, we performed footprinting assays coupled to SHAPE (selective 2′ hydroxyl acylation analyzed by primer extension) analysis. We have recently determined *tat1* and *tat2* mRNA folding using enzymatic and chemical probing and identified a highly conserved sequence-structure within MS RNA, TIM-TAM (Khoury et al., 2020). TIM-TAM forms the apical part of an irregular stem-loop structure SLS3_A__3_ that harbors the Tat start codon. TIM-TAM controls the timing and level of Tat translation during the early and late phases of infection, while promoting latent infection and virus reactivation. Footprinting assays were performed on RNP complexes formed by *tat2* transcript (Figure 5A) and recombinant proteins at three different [RNA]/[protein] ratios 5, 10, and 20. Normalized shape reactivities and probing data were used to determine the binding sites of SRP14, HMGB3, and PTB. At the lowest [RNA]/[protein] ratio, protections were mainly detected on SLS3_A__3_ including TIM-TAM and its bordering sequences. Strong protections were also observed in the 5′ untranslated region, more specifically on the TAR, PBS, and DIS elements (Figure 5B). Upon increasing SRP14, HMGB3, and PTB concentration, protections of TIM-TAM were reinforced and new ones were detected on the Tat start codon and in the vicinity of SA3. Altogether, these data are consistent with direct binding of SRP14, HMGB3 as well as PTB to MS RNA highlighting a potential role of these RNA binding proteins in controlling Tat expression during latent infection.

### SRP14 Reactivates HIV-1 Latently Infected Cells and Virus Production

**FIGURE 6:**
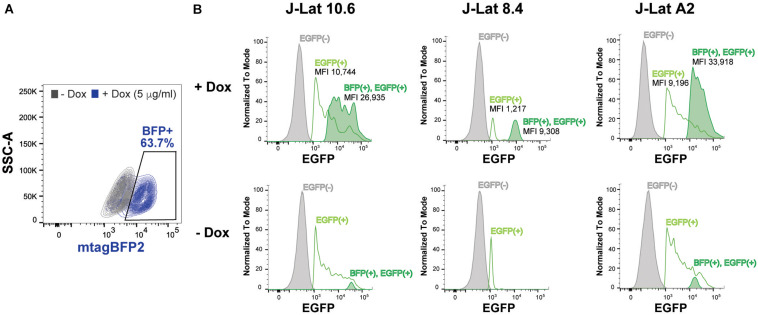
SRP14 expression reactivates latently infected T-cell lines. **(A)** J-Lat cells were transduced with SRP14-T2A-mtagBFP2 lentivector and cultured with (+Dox) or without (–Dox) doxycycline at 5 μg/mL. Cells were analyzed for mtagBFP2 expression by flow cytometry 72 h post-infection. **(B)** Histogram depicting EGFP^+^ expression in latently infected T-cells following transduction with SRP14-T2-mtagBFP2. Virus reactivation in J-Lat 10.6, 8.4 and A2 clones is shown by an increase in mean fluorescence intensity (MFI) of BFP^(+)^EGFP^(+)^ cells in comparison to EGFP^(–)^ and EGFP^(+)^ cells.

To assess the role of SRP14 in controlling latent infection, we tested the effect of its overexpression on virus reactivation in a T-cell line model of latent infection, using the J-Lat 10.6, 8.4 and A2 clones. J-Lats are Jurkat derived cells containing one stably integrated, but transcriptionally silenced full-length HIV-1 genome with GFP in place of the nef gene (Jordan et al., 2003). J-Lat cells were transduced with pInducer-SRP14-T2A-mtagBFP2 lentiviral vectors and cultured in the absence or presence of doxycycline (+Dox) for 2 days. Representative scatter plot highlighting mtagBFP2 expression following treatment of transduced cells with 5 μg/mL doxycycline is shown in Figure 6A. SRP14 expression was validated by western-blot analysis (data not shown). The different J-Lat clones exhibit variable levels of basal GFP expression. Upon doxycycline treatment, we observed an increase in GFP expression in all T-cell lines. Indeed, a significant increase in mean fluorescence intensity (MFI) was detected for BFP^+^GFP^+^ cells vs. GFP^+^ cells alone (Figure 6B).

**FIGURE 7:**
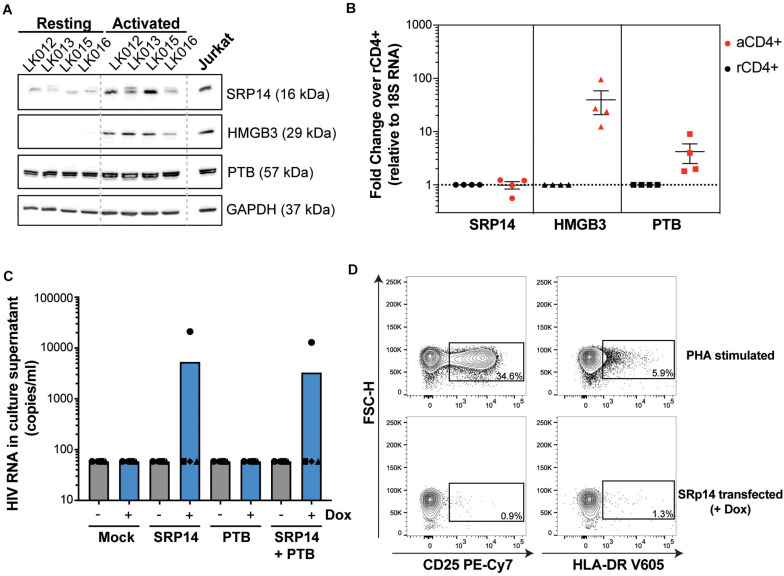
SRP14 and PTB expression induce HIV expression in primary CD4^+^ T-cells from patients on ART. **(A)** SRP14, HMGB3 and PTB protein expression in resting CD4^+^ T-cells isolated from patient under antiretroviral therapy (LK, leukophoresis) increase in response to T-cell stimulation by α-CD3/CD28 (1 μg/mL; 0.5 μg/mL). Expression of SRP14, HMGB3, and PTB was measured by western-blot in resting and activated CD4^+^ T-cells 72 h post-stimulation. GAPDH was used as a loading control and Jurkat protein lysate as a positive control for antibody detection of the various proteins. **(B)** SRP14, HMGB3, and PTB RNA levels were assessed by RT-qPCR and shown as fold change in activated (aCD4^+^) vs. resting CD4^+^ (rCD4^+^) after normalization on 18S RNA. **(C)** Effect of SRP14, HMGB3 and PTB overexpression following doxycycline (+Dox) treatment on HIV-1 RNA levels in the culture supernatant 48 h post-stimulation. **(D)** Plots depicting the expression of middle (CD25) and late (HLA-DR) activation markers on SRP14 transfected cells and PHA stimulated cells.

To determine whether differences in RNA binding protein levels in resting and activated CD4^+^ T-cells might be involved in the observed effect on HIV expression, we measured SRP14, HMGB3 and PTB protein and RNA levels in resting and α-CD3/CD28 stimulated CD4^+^ T-cells from patients living with HIV on ART. Western-blot analysis indicated low expression levels of SRP14 and HMGB3 in resting cells, and upon stimulation a 2.5- and 18.8-fold increase in SRP14 and HMGB3 protein level was detected, respectively (Figure 7A). Similar results were observed with RT-qPCR analysis of *HMGB3* mRNA levels between resting and activated CD4^+^ T-cells as we detected a 28.5-fold increase in *HMGB3* mRNA expression following CD4^+^ T-cell activation (Figure 7B). However, no changes in *SRP14* mRNA levels were seen in response to T-cell activation. Interestingly, while a 4.4-fold increase in *PTB* mRNA expression was observed, no significant change in PTB protein expression was detected following stimulation of CD4^+^ T-cells.

PTB was identified as an HIV RNA binding protein that induces virus reactivation and release of replication competent virus in resting CD4^+^ T-cells from patient on ART (Lassen et al., 2006). To examine whether SRP14 might also act as a positive factor for HIV-1 gene expression, resting CD4^+^ T-cells isolated from people living with HIV on ART were electroporated with SRP14 or PTB Dox-inducible expression constructs alone or in combination using an Amaxa nucleofector. After 48 h, virion release into the culture supernatant was assessed using an RT-ddPCR assay. One out of the four patient CD4^+^ T-cells electroporated with SRP14 and SRP14^+^ PTB presented an upregulation of virus production upon doxycycline treatment (Figure 7C). Virus production following SRP14 overexpression was not coupled with an increase in cell activation as only 0.9% and 1.3% of SRP14 transfected cells expressed middle (CD25) and late (HLA-DR) activation markers, respectively (Figure 7D).

## Discussion

**FIGURE 8:**
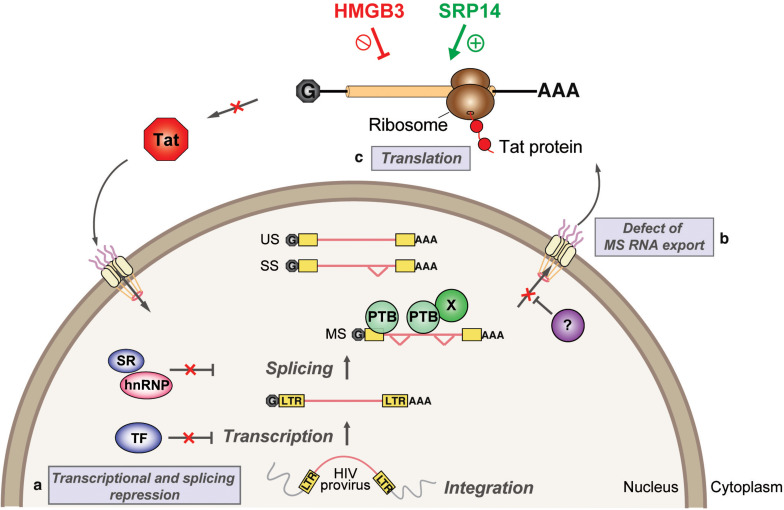
Model depicting SRP14 and HMGB3 roles in post-transcriptional regulation of HIV-1 gene expression in latently infected cells. Multiple factors have been implicated in the establishment and maintenance of latency including **(a)** transcriptional and splicing repression by sequestration of essential transcription factors (TF) and/or splicing factors; **(b)** defect in multiply spliced (MS) RNA export due to insufficient levels of Rev and/or RNA binding factors such as PTB (for polypyrimidine tract binding protein); and **(c)** aberrant localization of MS RNA in the nucleus imposes a block on translation. Due to its central role in transcriptional transactivation and production of full-length viral mRNAs, small stochastic changes in Tat expression, the absence or impairment of Tat function would favor latent infection. By targeting *tat* mRNA, SRP14 activates Tat translation and hence stimulates HIV replication, while HMGB3 protein inhibits Tat translation and consequently impedes HIV expression in resting CD4^+^ T cells.

We have characterized *tat* RNA:cellular protein interactions differentially expressed between productive and latent infection. Out of the 243 proteins identified by mass spectrometry, multiple cellular factors were investigated for their putative roles in the control of Tat expression and viral replication. A consistent effect on Tat expression and HIV-1 replication was exerted by both SRP14 and HMGB3, where SRP14 acts as a positive regulator of Tat expression and negative regulator of latent infection while HMGB3 acts as a negative regulator of Tat expression and negative regulator of productive infection (Figure 8). However, the exact mechanisms exerted by SRP14 and HMGB3 on the pathways of Tat expression have not been determined. It should be noted that knockdown of SRP14 and HMGB3 affected to a larger degree Tat expression in the IRES context. This suggests that SRP14 and HMGB3 proteins are acting through the Tat IRES pathway, by directly interacting with TIM-TAM or by acting as scaffolds for other RNA-binding proteins.

In a recent study, Yukl’s group identified HIV multiple splicing as a common block in three primary cell models of latent infection and in peripheral CD4^+^ T-cells isolated from HIV infected ART suppressed individuals (Moron-Lopez et al., 2020), confirming previous findings from the same group that showed a series of blocks to HIV proximal elongation, distal transcription/polyadenylation and splicing preventing HIV expression in CD4^+^ T-cells from blood of HIV infected patients on ART (Yukl et al., 2018). Prior studies have shown that MS RNA encoding Tat protein inhibits the establishment of HIV latency (Donahue et al., 2012). When present, Tat activates virus replication at a higher rate than any of the known LRAs (Razooky et al., 2015; Khoury et al., 2018b) by potently inducing HIV transcription and splicing (Khoury et al., 2018b). Moreover, by controlling its own production at the splicing (Jablonski et al., 2010) and translational levels (Charnay et al., 2009, Khoury et al., 2020), Tat acts as a switch for productive and latent HIV infection (Khoury et al., 2020).

siRNA knockdown of TOP2A in Sup T1 cells (Balakrishna et al., 2013), MAP4 in TZM-bl, HEK 293T and HeLa P4.2 cells (Brass et al., 2008; König et al., 2008; Gallo and Hope, 2012), HNRNPH1 in 293T cells (König et al., 2008), DDX1 in HeLa cells (Edgcomb et al., 2012) and HNRNPU in HeLa P4/R5 cells (Zhou et al., 2008) inhibit HIV-1 replication, corroborating our findings following shRNA KD of these proteins in Jurkat cells. Furthermore, an enrichment in mRNA processing proteins was observed in two previous HIV pull-down assays that used HIV-1 5′ leader sequence and unspliced RNA (Vallejos et al., 2011; Knoener et al., 2017). Although the pull-down methods and cell types used in both these studies were distinct, common proteins with our *tat* RNA pull-down assay were identified such as HNRNPL, HNRNPU, SRP14, and TERA that were isolated from HeLa cells arrested at G2/M with the HIV-1 5′UTRgag (Vallejos et al., 2011), as well as PPIA, GSTP1, STIP1, PHB, NUDC, FLNB, FUBP1, DEK, MAP4, CLIC1, and CD47 (Supplementary Table 4) identified from Jurkat cells infected with NL4-3 and unspliced HIV-1 RNA-cellular protein complexes (Knoener et al., 2017).

It should be noted that the large isoform of PTB, PTBP1, can overcome the nuclear retention of MS RNA in latently infected cells when overexpressed (Lassen et al., 2006). However, the levels detected in resting CD4^+^ T-cells from patients on ART were no different to activated CD4^+^ T-cells. Hence the specific isoform involved in Tat expression may be distinct to the isoform responsible for reversing aberrant accumulation of MS RNA. Further studies are required to determine the contribution of PTB to latency.

SRP14 (Signal Recognition Particle 14) is part of the signal recognition particle RNP complex, which functions by arresting the ribosome during translation so that secretory proteins can be correctly targeted to the endoplasmic reticulum (Weichenrieder et al., 2000; Lakkaraju et al., 2008). SRP14 together with SRP9 inhibits both cap- and IRES-dependent initiation. By binding to 40S ribosomal subunits, SRP9/14 prevents 48S complex formation hence interfering with the recruitment of mRNA to 40S subunits (Ivanova et al., 2015). HMGB3 (High Mobility Group Box 3) is thought to be a DNA-binding protein that can remodel chromatin structures and can also act as a nucleic acid sensor—these are putative functions inferred from its shared homology with other proteins in the HMG-Box family (Nemeth et al., 2003). An analysis of the entire mRNA bound proteome in HEK 293 cells, however, identified HMGB3 as a potential RNA-binding protein (Baltz et al., 2012) although to date, no specific RNA binding associated function has been reported for HMGB3. In the present study, SRP14 and HMGB3 appear to have a positive and inhibitory role on Tat IRES translation, respectively. To our knowledge, these findings are the first to link SRP14 and HMGB3 to HIV expression. The exact mechanisms of which require investigation.

Our findings highlight SRP14 and HMGB3 as potential targets of pharmaceutical intervention. This still does not present an ideal situation as, akin to LRAs used in the past, targeting of these cellular proteins do not confer specificity against latent HIV-1. These proteins, however, are unlikely to have an involvement in gene expression pathways as extensive as that of the epigenetic modifiers and proteins involved in the NFκB and NFAT pathways, and hence may only impact upon the expression of a limited array of cellular genes.

There are several limitations in this study that should be acknowledged. Given the large number of cells required to complete the pull-down assay, we chose J-Lat cells to use for the screening of Tat RNA binding proteins during latency given their transformed nature and homogeneity of the cell population. While latently infected T-cell lines are useful for screening, a common limitation of using T-cell lines is that the intrinsic cellular factors and environment in T-cell lines are very different to that of primary T-lymphocytes hence the cellular factors that influence HIV latency may differ between T-cell lines and primary CD4^+^ T-cells. Another caveat is the pull-down assay was performed on a mixed population of productively infected cells, as 100% reactivation of J-Lat cells is never achieved even after treatment with strong mitogens, including TNF-α. Virus expression from HIV latently infected T-cells could be sorted into two sub-populations; inducible (GFP^+^) and non-inducible proviruses (GFP−), following treatment with TNF-α. The reporter systems used in this study assess different stages of the Tat expression pathway. However, these assays do not allow evaluation of these stages independently of the others. Further validation studies are required to determine the exact mechanisms exerted by SRP14 and HMGB3 on the pathways of Tat expression. Finally, in this study we did not address whether SRP14 expression is sufficient to circumvent MS RNA retention in the nucleus of resting CD4^+^ T-cells, and whether it can induce production of replication competent virus. Further experiments are also required to test the effect of HMGB3 and SRP14 on *tat* mRNA export and/or stability.

Reactivation of HIV-1 can result from fluctuations in the levels of Tat protein (Weinberger et al., 2005; Khoury et al., 2020) as well as cellular factors involved in reinforcement of silent infection (Burnett et al., 2009). Hence, targeting Tat expression may serve as the basis for development of a more biologically relevant “shock and kill” strategy, a process that could lead to the discovery of an effective and durable functional cure for HIV-1.

## Data Availability Statement

The datasets presented in this study can be found in online repositories. The names of the repository/repositories and accession number(s) can be found below: PRIDE, PXD025782.

## Ethics Statement

The studies involving the use of blood samples from HIV negative donors were reviewed and approved by the Human Research and Ethics Committees from the University of Melbourne (15-09VIC-03 and 17-08VIC-01). All HIV-1 seronegative donors were recruited by the Red Cross Blood Bank (Melbourne, Australia) and provided written informed consent for the use of their blood products for the research. The use of blood samples from people living with HIV was approved by the Alfred Hospital (HREC214/15) for the study entitled Large volume peripheral blood mononuclear cells (PBMCs) collection by leukapheresis to define HIV persistence in HIV-infected adults. All participants provided informed consent and the protocol was approved by the local Institutional Review Board. The patients/participants provided their written informed consent to participate in this study.

## Author Contributions

GK, ML, and DP conceived and designed the study. GK, ML, and SR acquired the data. GK, ML, SS, and DP analyzed and interpreted the data. JM, AP, and SL provided resources. GK and ML drafted the manuscript. All authors read and approved the final version of the manuscript.

## Conflict of Interest

The authors declare that the research was conducted in the absence of any commercial or financial relationships that could be construed as a potential conflict of interest.

## Supplementary Material

Supplementary Figure 1 GO Biological Process analysis of the full set of proteins detected by mass spectrometry. List of proteins that were uniquely detected in the pull-down assay using untreated J-Lat 6.3 **(A)** or TNF-α activated **(B)** lysates were analyzed through *Enrichr* and overrepresented GO terms are depicted in the bar graphs. Examples of proteins belonging to the top classes are listed.

Supplementary Figure 2 Protein and mRNA profiles of KD RFP^+^ Jurkat cells. **(A)** Representative histograms of RFP^+^ shRNA transduced Jurkats (bulk cells) where SRP14, HMGB3 or PTB were targeted for knockdown. **(B)** Representative western blots showing knockdown in protein levels of the three genes of interest, SRP14, HMGB3, PTB in their respective bulk shRNA-generated cell lines. Numbers below the blots show relative amounts of proteins in comparison to untransduced Jurkats. **(C)** Expression of RFP in bulk or single clone Jurkat cells targeted for knockdown of SRP14, HMGB3 or PTB. **(D)** Fold change in SRP14, HMGB3 and PTB mRNA levels in their respective bulk shRNA-generated cell lines or single clones compared to untreated Jurkat cells as determined by RT-qPCR.

Supplementary Figure 3 R7GEmTB reporter virus label latently infected cells. Jurkat and CD4^+^ T-cells were infected with R7GEmTB dual color reporter virus and subjected to confocal microscopy to assess the infection phenotype. GFP^+^ and GFP^+^ BFP^+^ cells, representative of productive infection are shown in green and cyan, while BFP^+^ cells representative of latent infection are shown in blue.

Supplementary Figure 4 Western-blot analysis of RNP complexes. Analysis of the protein content of the RNP complexes formed on *tat* mRNA by western-blot using antibodies directed against SRP14 and HMGB3. MBP-MS2 was used as a loading control (ponceau stain). JL− and JL+ represent total lysates prepared from J-Lat 6.3 left untreated or treated with TNF-α, respectively.

Supplementary Table 1 Participant demographics.

Supplementary Table 2 Primers and probes used in this study. The number, sequence, and usage of each primer are given. Restriction sites are underlined, start and stop codons are in boldfaces.

Supplementary Table 3 List of protein hits selected for follow-up analysis. Summary of the gene ID, uniprot, gene symbol, protein name, function, mass (in kDa), and protein length (in amino acid, aa) are indicated.

Supplementary Table 4 List of proteins identified by affinity purification coupled to mass spectrometry in the RNP complexes formed with 5′UTRtat1-Tat-MS2 and Tat-MS2 RNAs. The number of peptides identified for each bait (5′UTRtat-Tat-MS2 and Tat-MS2) and prey (protein) as well as their fold-enrichment in resting and activated cells are indicated.

## References

[B1] Ait-Ammar A. Kula A. Darcis G. Verdikt R. De Wit S. Gautier V. (2020). Current status of latency reversing agents facing the heterogeneity of HIV-1 cellular and tissue reservoirs. *Front. Microbiol.* 10:3060. 10.3389/fmicb.2019.03060 32038533 PMC6993040

[B2] Anderson I. Low J. S. Weston S. Weinberger M. Zhyvoloup A. Labokha A. A. (2014). Heat shock protein 90 controls HIV-1 reactivation from latency. *Proc. Natl. Acad. Sci. U S A.* 111 E1528–E1537. 24706778 10.1073/pnas.1320178111 PMC3992654

[B3] Balakrishna S. L. Satyanarayana N. Kondapi A. K. (2013). Involvement of human topoisomerase II isoforms in HIV-1 reverse transcription. *Arch. Biochem. Biophys.* 532 91–102. 10.1016/j.abb.2013.01.010 23399433

[B4] Baltz A. G. Munschauer M. Schwanhausser B. Vasile A. Murakawa Y. Schueler M. (2012). The mRNA-bound proteome and its global occupancy profile on protein-coding transcripts. *Mol. Cell.* 46 674–690. 10.1016/j.molcel.2012.05.021 22681889

[B5] Bar A. Marchand V. Khoury G. Dreumont N. Mougin A. Robas N. (2011). Structural and functional analysis of the Rous Sarcoma virus negative regulator of splicing and demonstration of its activation by the 9G8 SR protein. *Nucleic Acids Res.* 39 3388–3403. 10.1093/nar/gkq1114 21183462 PMC3082916

[B6] Brass A. L. Dykxhoorn D. M. Benita Y. Yan N. Engelman A. Xavier R. J. (2008). Identification of host proteins required for HIV infection through a functional genomic screen. *Science* 319 921–926. 10.1126/science.1152725 18187620

[B7] Burnett J. C. Miller-Jensen K. Shah P. S. Arkin A. P. Schaffer D. V. (2009). Control of stochastic gene expression by host factors at the HIV promoter. *PLoS Pathog.* 5:e1000260. 10.1371/journal.ppat.1000260 19132086 PMC2607019

[B8] Calvanese V. Chavez L. Laurent T. Ding S. Verdin E. (2013). Dual-color HIV reporters trace a population of latently infected cells and enable their purification. *Virology* 446 283–292. 10.1016/j.virol.2013.07.037 24074592 PMC4019006

[B9] Charnay N. Ivanyi-Nagy R. Soto-Rifo R. Ohlmann T. Lopez-Lastra M. Darlix J. L. (2009). Mechanism of HIV-1 Tat RNA translation and its activation by the Tat protein. *Retrovirology* 6:74. 10.1186/1742-4690-6-74 19671151 PMC2739156

[B10] Chiu Y. L. Coronel E. Ho C. K. Shuman S. Rana T. M. (2001). HIV-1 Tat protein interacts with mammalian capping enzyme and stimulates capping of TAR RNA. *J. Biol. Chem.* 276 12959–12966. 10.1074/jbc.m007901200 11278368

[B11] Chiu Y.-L. Ho C. K. Saha N. Schwer B. Shuman S. Rana T. M. (2002). Tat stimulates cotranscriptional capping of HIV mRNA. *Mol. Cell.* 10 585–597. 10.1016/s1097-2765(02)00630-5 12408826

[B12] Clouse K. A. Powell D. Washington I. Poli G. Strebel K. Farrar W. (1989). Monokine regulation of human immunodeficiency virus-1 expression in a chronically infected human T cell clone. *J. Immunol.* 142 431–438. 2463307

[B13] Cvitkovic I. Jurica M. S. (2013). Spliceosome database: a tool for tracking components of the spliceosome. *Nucleic Acids Res.* 41 132–141. 10.1093/nar/gks999 PMC3531166 23118483

[B14] Deckert J. Hartmuth K. Boehringer D. Behzadnia N. Will C. L. Kastner B. (2006). Protein composition and electron microscopy structure of affinity-purified human spliceosomal B complexes isolated under physiological conditions. *Mol. Cell Biol.* 26 5528–5543. 10.1128/mcb.00582-06 16809785 PMC1592722

[B15] Deeks S. G. Lewin S. R. Ross A. L. Ananworanich J. Benkirane M. Cannon P. (2016). International AIDS Society global scientific strategy: Towards an HIV cure 2016. *Nat. Med.* 22 839–850. 27400264 10.1038/nm.4108 PMC5322797

[B16] Donahue D. A. Kuhl B. D. Sloan R. D. Wainberg M. A. (2012). The viral protein Tat can inhibit the establishment of HIV-1 latency. *J. Virol.* 86 3253–3263. 10.1128/jvi.06648-11 22238306 PMC3302319

[B17] Edgcomb S. P. Carmel A. B. Naji S. Ambrus-Aikelin G. Reyes J. R. Saphire A. C. S. (2012). DDX1 is an RNA-dependent ATPase involved in HIV-1 Rev function and virus replication. *J. Mol. Biol.* 415 61–74. 10.1016/j.jmb.2011.10.032 22051512 PMC3249508

[B18] Folks T. M. Clouse K. A. Justement J. Rabson A. Duh E. Kehrl J. H. (1989). Tumor necrosis factor alpha induces expression of human immunodeficiency virus in a chronically infected T-cell clone. *Proc. Natl. Acad. Sci. U S A* 86 2365–2368. 10.1073/pnas.86.7.2365 2784570 PMC286913

[B19] Gallo D. E. Hope T. J. (2012). Knockdown of MAP4 and DNAL1 produces a post-fusion and pre-nuclear translocation impairment in HIV-1 replication. *Virology.* 422 13–21. 10.1016/j.virol.2011.09.015 22018492 PMC3732191

[B20] Ivanova E. Berger A. Scherrer A. Alkalaeva E. Strub K. (2015). Alu RNA regulates the cellular pool of active ribosomes by targeted delivery of SRP9/14 to 40S subunits. *Nucleic Acids Res.* 43 2874–2887. 10.1093/nar/gkv048 25697503 PMC4357698

[B21] Jablonski J. A. Amelio A. L. Giacca M. Caputi M. (2010). The transcriptional transactivator Tat selectively regulates viral splicing. *Nucleic Acids Res.* 38 1249–1260. 10.1093/nar/gkp1105 19966273 PMC2831323

[B22] Jäger S. Cimermancic P. Gulbahce N. Johnson J. R. McGovern K. E. Clarke S. C. (2011). Global landscape of HIV-human protein complexes. *Nature* 481 365–370. 22190034 10.1038/nature10719 PMC3310911

[B23] Jordan A. Bisgrove D. Verdin E. (2003). HIV reproducibly establishes a latent infection after acute infection of T cells in vitro. *EMBO J.* 22 1868–1877. 10.1093/emboj/cdg188 12682019 PMC154479

[B24] Karabiber F. McGinnis J. L. Favorov O. V. Weeks K. M. (2013). QuShape: rapid, accurate, and best-practices quantification of nucleic acid probing information, resolved by capillary electrophoresis. *RNA* 19 63–73. 10.1261/rna.036327.112 23188808 PMC3527727

[B25] Khoury G. Darcis G. Lee M. Y. Bouchat S. Van Driessche B. Purcell D. F. (2018a). *The Molecular Biology of HIV Latency. In: HIV vaccine and cure - The Path Towards Finding an Effective Cure and Vaccine.* Cham: Springer Nature. 10.1007/978-981-13-0484-2_8 30030794

[B26] Khoury G. Mota T. M. Li S. Tumpach C. Lee M. Y. Jacobson J. (2018b). HIV latency reversing agents act through Tat post translational modifications. *Retrovirology* 15:36. 10.1186/s12977-018-0421-6 PMC5948896 29751762

[B27] Khoury G. Mackenzie C. Ayadi L. Lewin S. R. Branlant C. Purcell D. F. J. (2020). Tat IRES modulator of tat mRNA (TIM-TAM): a conserved RNA structure that controls Tat expression and acts as a switch for HIV productive and latent infection. *Nucleic Acids Res.* 48 2643–2660. 10.1093/nar/gkz1181 31875221 PMC7049722

[B28] Knoener R. A. Becker J. T. Scalf M. Sherer N. M. Smith L. M. (2017). Elucidating the in vivo interactome of HIV-1 RNA by hybridization capture and mass spectrometry. *Sci. Rep.* 7:16965. 10.1038/s41598-017-16793-5 PMC5717263 29208937

[B29] König R. Zhou Y. Elleder D. Diamond T. L. Bonamy G. M. C. Irelan J. T. (2008). Global analysis of host-pathogen interactions that regulate early-stage HIV-1 Replication. *Cell* 135 49–60. 10.1016/j.cell.2008.07.032 18854154 PMC2628946

[B30] Krueger B. J. Varzavand K. Cooper J. J. Price D. H. (2010). The mechanism of release of P-TEFb and HEXIM1 from the 7SK snRNP by viral and cellular activators includes a conformational change in 7SK. *PLoS One.* 5:e12335. 10.1371/journal.pone.0012335 20808803 PMC2925947

[B31] Lakkaraju A. K. Mary C. Scherrer A. Johnson A. E. Strub K. (2008). SRP keeps polypeptides translocation-competent by slowing translation to match limiting ER-targeting sites. *Cell* 133 440–451. 10.1016/j.cell.2008.02.049 18455985 PMC2430734

[B32] Lassen K. G. Ramyar K. X. Bailey J. R. Zhou Y. Siliciano R. F. (2006). Nuclear retention of multiply spliced HIV-1 RNA in resting CD4+ T cells. *PLoS Pathog.* 2:e68. 10.1371/journal.ppat.0020068 16839202 PMC1487174

[B33] Liu J. Henao-Mejia J. Liu H. Zhao Y. He J. J. (2011). Translational regulation of HIV-1 replication by HIV-1 rev cellular cofactors Sam68, eIF5A, hRIP, and DDX3. *J. Neuroimmun. Pharmacol.* 6 308–321. 10.1007/s11481-011-9265-8 21360055

[B34] Lozano G. Martínez-Salas E. (2015). Structural insights into viral IRES-dependent translation mechanisms. *Curr. Opin. Virol.* 12 113–120. 10.1016/j.coviro.2015.04.008 26004307

[B35] Maenner S. Blaud M. Fouillen L. Savoye A. Marchand V. Dubois A. (2010). 2-D structure of the A region of Xist RNA and its implication for PRC2 association. *PLoS Biol.* 8:e1000276. 10.1371/journal.pbio.1000276 20052282 PMC2796953

[B36] Meerbrey K. L. Hu G. Kessler J. D. Roarty K. Li M. Z. Fang J. E. (2011). The pINDUCER lentiviral toolkit for inducible RNA interference in vitro and in vivo. *Proc. Natl. Acad. Sci. U S A.* 108 3665–3670. 10.1073/pnas.1019736108 21307310 PMC3048138

[B37] Monette A. Ajamian L. Lopez-Lastra M. Mouland A. J. (2009). Human immunodeficiency virus type 1 (HIV-1) induces the cytoplasmic retention of heterogeneous nuclear ribonucleoprotein A1 by disrupting nuclear import: implications for HIV-1 gene expression. *J. Biol. Chem.* 284 31350–31362. 19737937 10.1074/jbc.M109.048736 PMC2781532

[B38] Moron-Lopez S. Telwatte S. Sarabia I. Battivelli E. Montano M. Macedo A. B. (2020). Human splice factors contribute to latent HIV infection in primary cell models and blood CD4+ T cells from ART-treated individuals. *PLoS Pathog.* 16:e1009060. 10.1371/journal.ppat.1009060 33253324 PMC7728277

[B39] Mortimer S. A. Weeks K. M. (2007). A fast-acting reagent for accurate analysis of RNA secondary and tertiary structure by SHAPE chemistry. *J. Am. Chem. Soc.* 129 4144–4145. 10.1021/ja0704028 17367143

[B40] Muniz L. Egloff S. Ughy B. Jády B. E. Kiss T. (2010). Controlling cellular P-TEFb activity by the HIV-1 transcriptional transactivator tat. *PLoS Pathog.* 6:e1001152. 10.1371/journal.ppat.1001152 20976203 PMC2954905

[B41] Nemeth M. J. Curtis D. J. Kirby M. R. Garrett-Beal L. J. Seidel N. E. Cline A. P. (2003). Hmgb3: an HMG-box family member expressed in primitive hematopoietic cells that inhibits myeloid and B-cell differentiation. *Blood* 102 1298–1306. 10.1182/blood-2002-11-3541 12714519

[B42] Nguyen W. Jacobson J. Jarman K. E. Jousset Sabroux H. Harty L. McMahon J. (2019). Identification of 5-substituted 2-acylaminothiazoles that activate Tat mediated transcription in HIV-1 latency models. *J. Med. Chem.* 62 5148–5175. 10.1021/acs.jmedchem.9b00462 30973727

[B43] Ott M. Geyer M. Zhou Q. (2011). The control of HIV transcription: keeping RNA polymerase II on track. *Cell Host. Microb.* 10 426–435. 10.1016/j.chom.2011.11.002 22100159 PMC3478145

[B44] Pasternak A. O. Berkhout B. (2018). What do we measure when we measure cell-associated HIV RNA. *Retrovirology* 15:13. 10.1186/s12977-018-0397-2 PMC5789533 29378657

[B45] Perez-Riverol Y. Csordas A. Bai J. Bernal-Llinares M. Hewapathirana S. Kundu D. J. (2019). The PRIDE database and related tools and resources in 2019: improving support for quantification data. *Nucleic Acids Res.* 47 D442–D450. 30395289 10.1093/nar/gky1106 PMC6323896

[B46] Ping Y.-H. Rana T. M. (2001). DSIF and NELF Interact with RNA Polymerase II Elongation Complex and HIV-1 Tat Stimulates P-TEFb-mediated Phosphorylation of RNA Polymerase II and DSIF during Transcription Elongation ^∗^. *J. Biol. Chem.* 276 12951–12958. 10.1074/jbc.m006130200 11112772

[B47] Razooky B. S. Pai A. Aull K. Rouzine I. M. Weinberger L. S. (2015). A hardwired HIV latency program. *Cell* 160 990–1001. 10.1016/j.cell.2015.02.009 25723172 PMC4395878

[B48] Rivas-Aravena A. Ramdohr P. Vallejos M. Valiente-Echeverría F. Dormoy-Raclet V. Rodríguez F. (2009). The Elav-like protein HuR exerts translational control of viral internal ribosome entry sites. *Virology.* 392 178–185. 10.1016/j.virol.2009.06.050 19647848

[B49] Ropers D. Ayadi L. Gattoni R. Jacquenet S. Damier L. Branlant C. (2004). Differential effects of the SR proteins 9G8, SC35, ASF/SF2, and SRp40 on the utilization of the A1 to A5 splicing sites of HIV-1 RNA. *J. Biol. Chem.* 279 29963–29973. 10.1074/jbc.m404452200 15123677

[B50] Saleh S. Wightman F. Ramanayake S. Alexander M. Kumar N. Khoury G. (2011). Expression and reactivation of HIV in a chemokine induced model of HIV latency in primary resting CD4+ T cells. *Retrovirology* 8:80. 10.1186/1742-4690-8-80 21992606 PMC3215964

[B51] Saliou J. M. Bourgeois C. F. Ayadi-Ben Mena L. Ropers D. Jacquenet S. Marchand V. (2009). Role of RNA structure and protein factors in the control of HIV-1 splicing. *Front. Biosci.* 14 2714–2729. 10.2741/3408 19273230

[B52] Sarzotti-Kelsoe M. Bailer R. T. Turk E. Lin C. L. Bilska M. Greene K. M. (2014). Optimization and validation of the TZM-bl assay for standardized assessments of neutralizing antibodies against HIV-1. *J. Immunol. Methods.* 409 131–146. 10.1016/j.jim.2013.11.022 24291345 PMC4040342

[B53] Schapira M. Raaka B. M. Das S. Fan L. Totrov M. Zhou Z. (2003). Discovery of diverse thyroid hormone receptor antagonists by high-throughput docking. *Proc. Natl. Acad. Sci. U S A.* 100 7354–7359. 10.1073/pnas.1131854100 12777627 PMC165879

[B54] Spina C. A. Anderson J. Archin N. M. Bosque A. Chan J. Famiglietti M. (2013). An in-depth comparison of latent HIV-1 reactivation in multiple cell model systems and resting CD4+ T cells from aviremic patients. *PLoS Pathog.* 9:e1003834. 10.1371/journal.ppat.1003834 24385908 PMC3873446

[B55] Vallejos M. Deforges J. Plank T. D. Letelier A. Ramdohr P. Abraham C. G. (2011). Activity of the human immunodeficiency virus type 1 cell cycle-dependent internal ribosomal entry site is modulated by IRES trans-acting factors. *Nucleic Acids Res.* 39 6186–6200. 10.1093/nar/gkr189 21482538 PMC3152342

[B56] Weichenrieder O. Wild K. Strub K. Cusack S. (2000). Structure and assembly of the Alu domain of the mammalian signal recognition particle. *Nature* 408 167–173. 10.1038/35041507 11089964

[B57] Weinberger L. S. Burnett J. C. Toettcher J. E. Arkin A. P. Schaffer D. V. (2005). Stochastic gene expression in a lentiviral positive-feedback loop: HIV-1 Tat fluctuations drive phenotypic diversity. *Cell* 122 169–182. 10.1016/j.cell.2005.06.006 16051143

[B58] Yukl S. Pillai S. Li P. Chang K. Pasutti W. Ahlgren C. (2009). Latently-infected CD4+ T cells are enriched for HIV-1 Tat variants with impaired transactivation activity. *Virology* 387 98–108. 10.1016/j.virol.2009.01.013 19268337 PMC4474533

[B59] Yukl S. A. Kaiser P. Kim P. Telwatte S. Joshi S. K. Vu M. (2018). HIV latency in isolated patient CD4+T cells may be due to blocks in HIV transcriptional elongation, completion, and splicing. *Sci. Transl. Med.* 10:eaa9927. 10.1126/scitranslmed.aap9927 PMC5959841 29491188

[B60] Zerbato J. M. Khoury G. Zhao W. Gartner M. J. Pascoe R. D. Rhodes A. (2021). Multiply spliced HIV RNA is a predictive measure of virus production ex vivo and in vivo following reversal of HIV latency. *EBioMedicine* 65:103241. 10.1016/j.ebiom.2021.103241 33647768 PMC7920823

[B61] Zerbato J. M. Purves H. V. Lewin S. R. Rasmussen T. A. (2019). Between a shock and a hard place: challenges and developments in HIV latency reversal. *Curr. Opin. Virol.* 38 1–9. 10.1016/j.coviro.2019.03.004 31048093 PMC6819240

[B62] Zhou H. Xu M. Huang Q. Gates A. T. Zhang X. D. Castle J. C. (2008). Genome-scale RNAi screen for host factors required for HIV replication. *Cell. Host. Microb.* 4 495–504. 10.1016/j.chom.2008.10.004 18976975

