# Supplementary figures and images for: The RNA-Binding Proteins SRP14 and HMGB3 Control HIV-1 Tat mRNA Processing and Translation During HIV-1 Latency
Source: Front Genet. 2021 Jun 14;12:680725. doi: 10.3389/fgene.2021.680725 (PMC8236859; doi:10.3389/fgene.2021.680725)

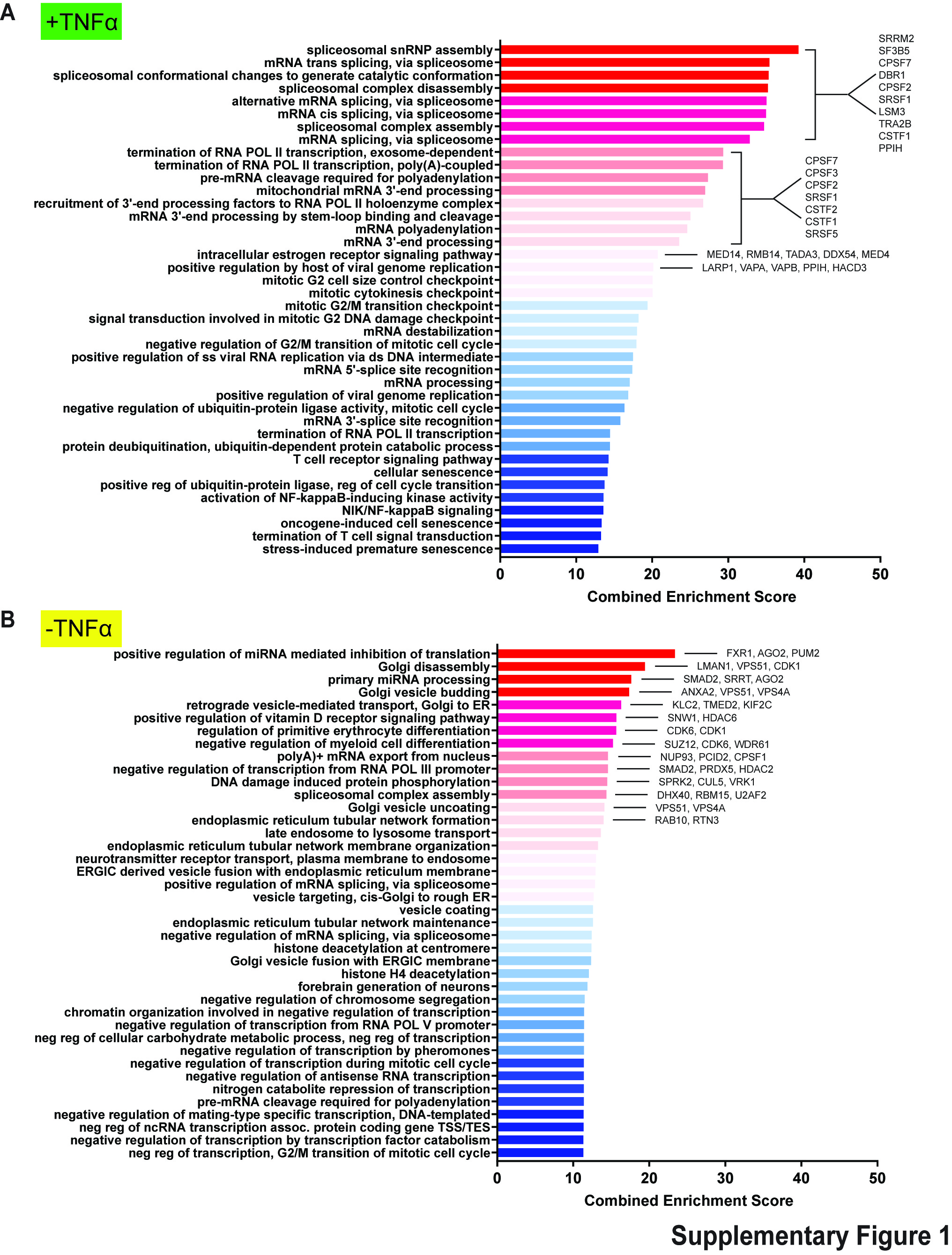

Supplement: Supplementary Figure 1 — GO Biological Process analysis of the full set of proteins detected by mass spectrometry. List of proteins that were uniquely detected in the pull-down assay using untreated J-Lat 6.3 (A) or TNF-α activated (B) lysates were analyzed through Enrichr and overrepresented GO terms are depicted in the bar graphs. Examples of proteins belonging to the top classes are listed. [file Image_1.jpg]

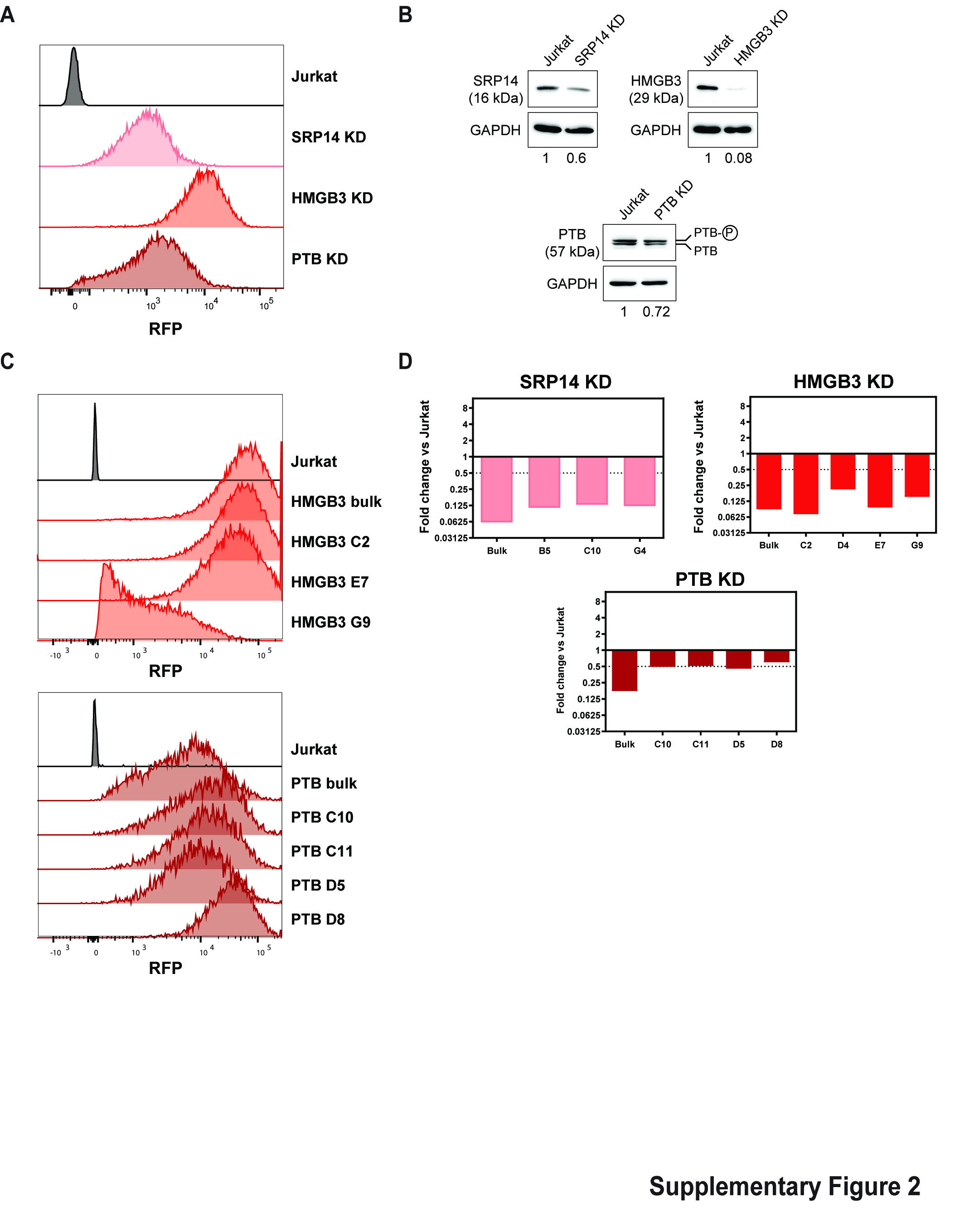

Supplement: Supplementary Figure 2 — Protein and mRNA profiles of KD RFP+ Jurkat cells. (A) Representative histograms of RFP+ shRNA transduced Jurkats (bulk cells) where SRP14, HMGB3 or PTB were targeted for knockdown. (B) Representative western blots showing knockdown in protein levels of the three genes of interest, SRP14, HMGB3, PTB in their respective bulk shRNA-generated cell lines. Numbers below the blots show relative amounts of proteins in comparison to untransduced Jurkats. (C) Expression of RFP in bulk or single clone Jurkat cells targeted for knockdown of SRP14, HMGB3 or PTB. (D) Fold change in SRP14, HMGB3 and PTB mRNA levels in their respective bulk shRNA-generated cell lines or single clones compared to untreated Jurkat cells as determined by RT-qPCR. [file Image_2.jpg]

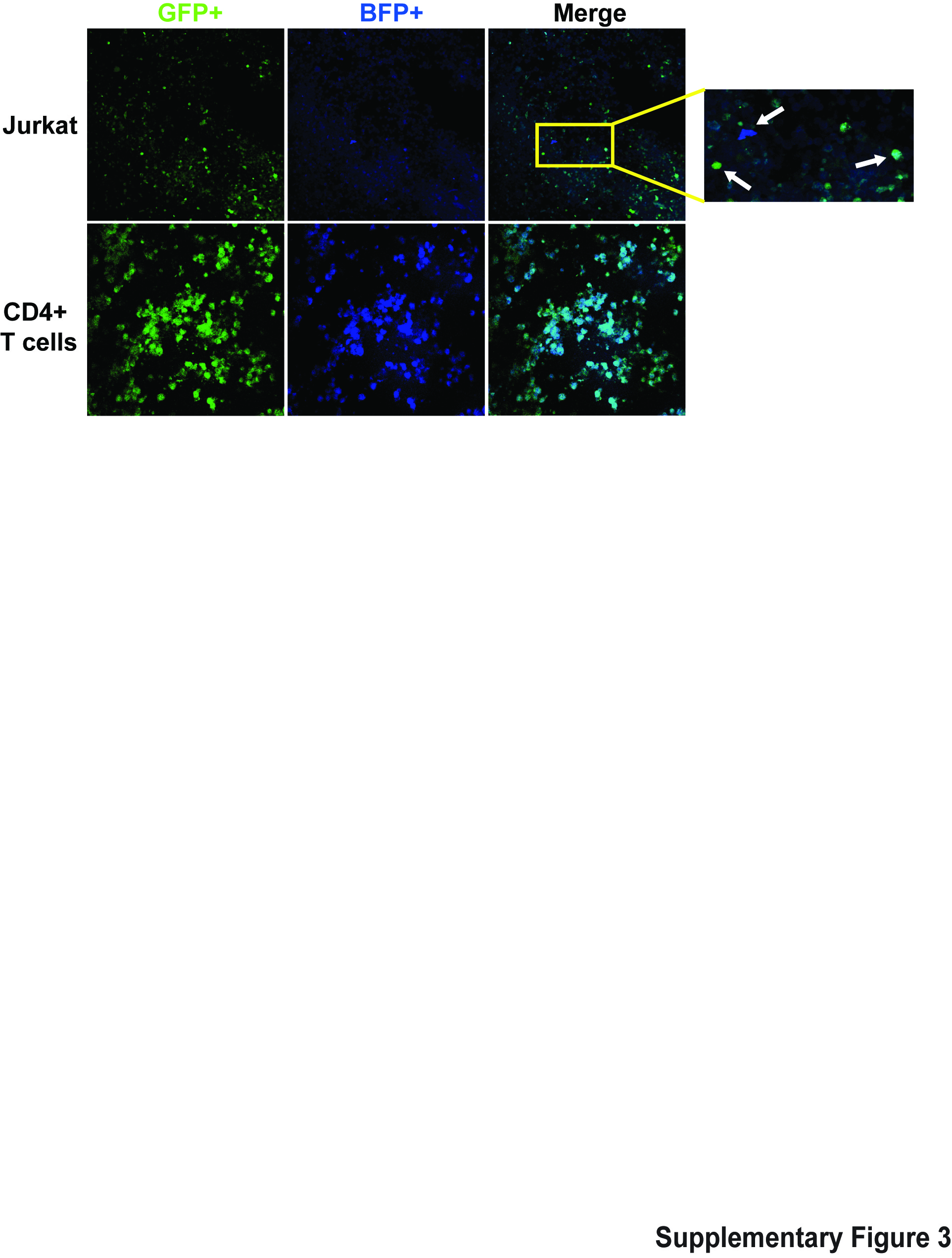

Supplement: Supplementary Figure 3 — R7GEmTB reporter virus label latently infected cells. Jurkat and CD4+ T-cells were infected with R7GEmTB dual color reporter virus and subjected to confocal microscopy to assess the infection phenotype. GFP+ and GFP+ BFP+ cells, representative of productive infection are shown in green and cyan, while BFP+ cells representative of latent infection are shown in blue. [file Image_3.jpg]

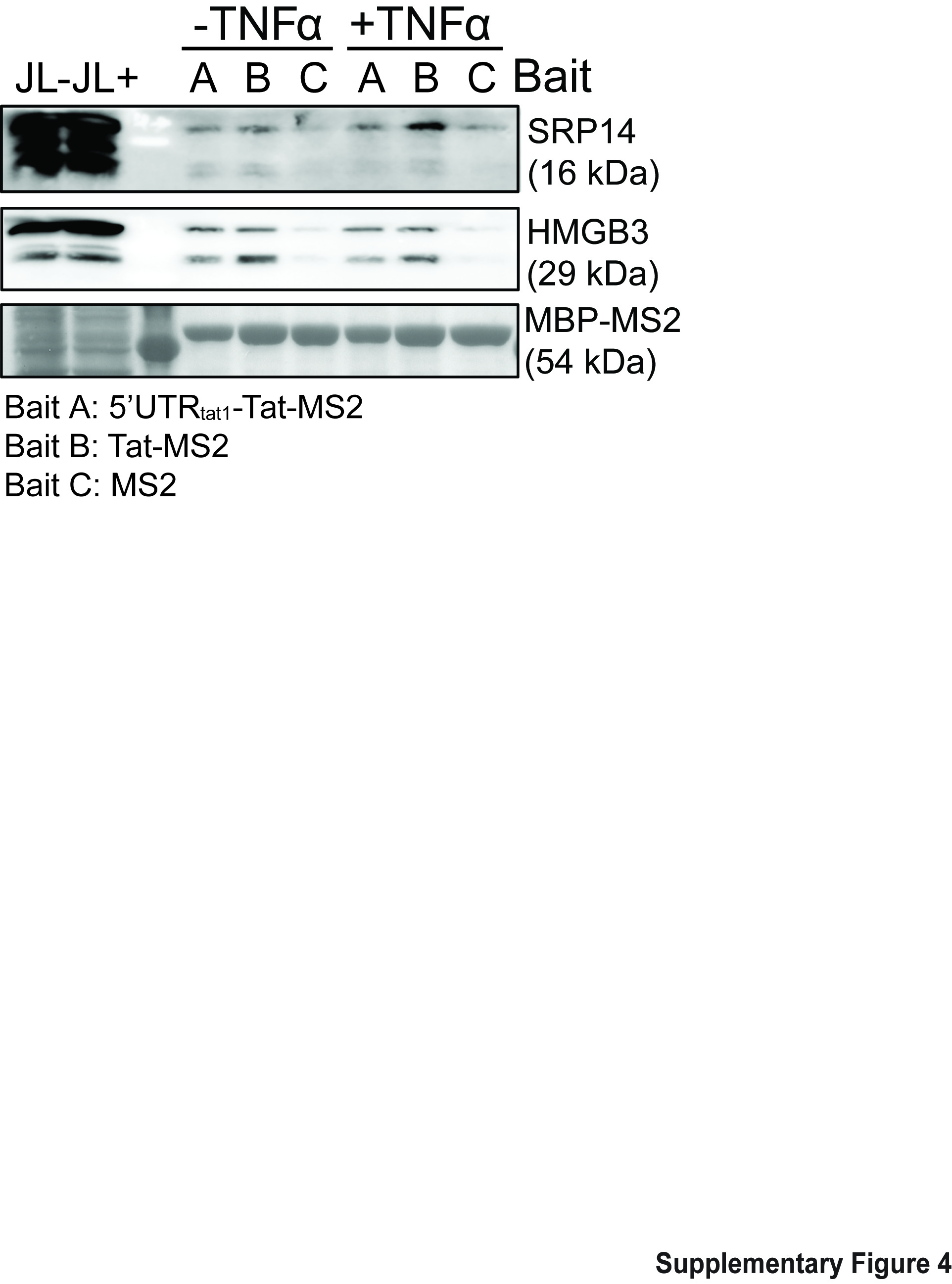

Supplement: Supplementary Figure 4 — Western-blot analysis of RNP complexes. Analysis of the protein content of the RNP complexes formed on tat mRNA by western-blot using antibodies directed against SRP14 and HMGB3. MBP-MS2 was used as a loading control (ponceau stain). JL− and JL+ represent total lysates prepared from J-Lat 6.3 left untreated or treated with TNF-α, respectively. [file Image_4.jpg]
