# Supplementary material for: The RNA-Binding Proteins SRP14 and HMGB3 Control HIV-1 Tat mRNA Processing and Translation During HIV-1 Latency
Source: Front Genet. 2021 Jun 14;12:680725. doi: 10.3389/fgene.2021.680725 (PMC8236859; doi:10.3389/fgene.2021.680725)
Supplement: Supplementary Table 1 — Participant demographics. [file Data_Sheet_1.zip › Suppl. Tables 1 & 2.DOCX]

**
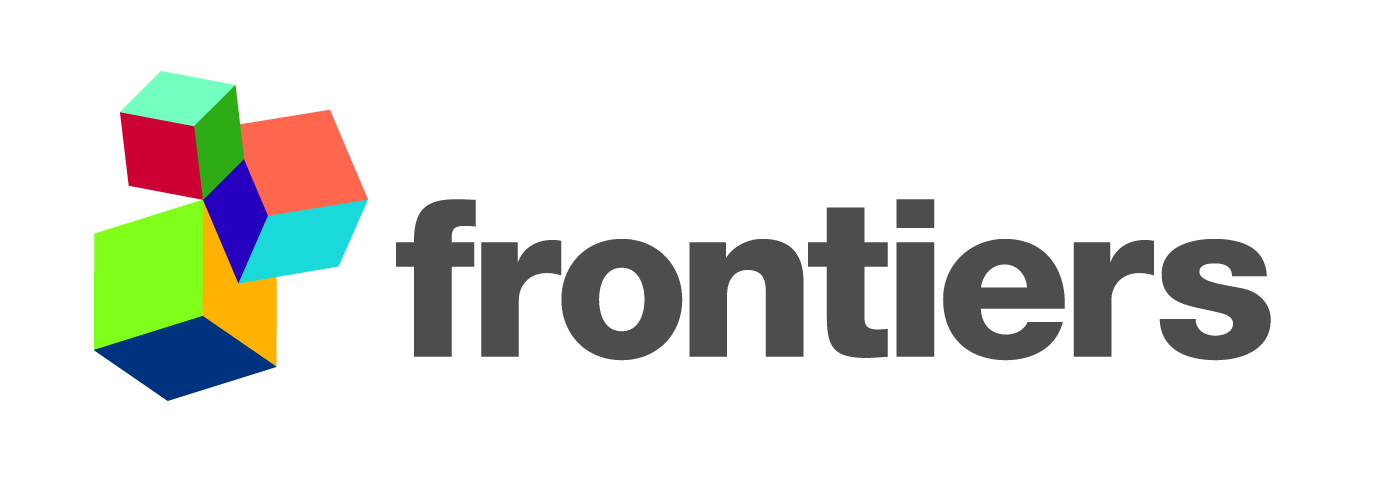
**

Supplementary Material

# Supplementary Tables

| **Participant ID** | **Age (years)** | **Sex (M or F)** | **Duration on ART (years)** | **CD4 T cell count (cells/µL)** | **Viral load (copies/mL)** |
| --- | --- | --- | --- | --- | --- |
| LK12 | 57 | M | 14.7 | 744 | < 20 |
| LK13 | 58 | M | >1 | 520 | < 20 |
| LK15 | 43 | M | 10.2 | 681 | < 20 |
| LK16 | 67 | M | 10.9 | 534 | < 20 |
| Median | 57.5 |  | 10.5 | 607.5 | < 20 |

**Supplementary Table 1:** Participant demographics.

| **Primer Number** | **Sequence (5’ – 3’)** | **Position** | **Utilisation** |
| --- | --- | --- | --- |
| **SHAPE analysis** | | | |
| **Odp3102** | 5’-GGCAATGAAAGCAAC-3’ | 5500-5514  (Bru) | Primer extension |
| **Expression constructs** | | | |
| **Odp2683** | 5’-ATAAAGCTAGCGGTCTCTCTGG-3’ |  | Amplification of  5’UTRtat1-Tat  *(NheI, used with Odp2690)* |
| **Odp2690** | 5’-ATAAAGAATTC**CTA**TTCCTTCGGGCCTGTCGGGTCCCC-3’ |  | Amplification of  5’UTRtat1-Tat; Tat  *(EcoRI, stop codon, used with Odp2683 and Odp2691)* |
| **Odp2691** | 5’-ATAAAGCTAGC**ATG**GAGCCAGTAGATCCTAGACTAGAG-3’ |  | Amplification of Tat  *(NheI, start codon,*  *used with Odp2690)* |
| **Odp2763** | 5’-ATAAAGGATCC**CGTACACCATCAGGGTACG**  gatatcccatgg**CGTACACCATCAGGG**-3’ |  | Amplification of 3x MS2  *(BamHI, used with Odp2764)* |
| **Odp2764** | 5’-agatctactagt**CGTACCCTGATGGTGTACG**ccatgg-3’ |  | Amplification of 3x MS2  *(used with Odp2763)* |
| **Odp2765** | 5’-ATAAAGGTACC**CGTACCCTGATGGTGTACG**agatctactagt  **CGTACCCTGAT**-3’ |  | Cloning of 3x MS2  *(KpnI)* |
| **Odp3566** | 5’-ATAAAACCGGTATGGGCAGCAGC**CATCATCATCATCATCAC**  AGC-3’ |  | Amplification of SRP14, HMGB3, PTB  *(AgeI, His6 tag)* |
| **Odp3715** | 5’-ATAAAAAGCTT**TTA**CTGTGCTGCTGTTGCTGCTGTTG-3’ | 386-411 | Amplification of SRP14  *(HindIII, stop codon)* |
| **Odp3716** | 5’-ATAAAAAGCTT**TTA**TTCATCCTCTTCTTCCTCTTCCTCCTC-3’ | 574-603 | Amplification of HMGB3  *(HindIII, stop codon)* |
| **Odp3717** | 5’-ATAAAAAGCTT**CTA**GATGGTGGACTTGGAGAAGGAG-3’ | 1650-1674 | Amplification of PTBP1  *(HindIII, stop codon)* |
| **Odp3713** | 5’-**AATCCTGGCCCA**ATCGATAGCGAACTGATCAAAGAG-3’ | 4-21 | Amplification of mtagBFP2  *(T2A, ClaI)* |
| **Odp3517** | 5’-ATAAAACCGGT**TTA**ATTCAGTTTATGACCCAGCTTG-3’ | 678-702 | Amplification of mtagBFP2  *(AgeI, stop codon)* |
| **HIV mRNA levels quantification by ddPCR** | | | |
| **Odp3063** | 5’-GCACTTTAAATTTTCCCATTAGTCCTA-3’ | 2536-2562  (pNL4-3) | ddPCR US Fw (*pol* RNA) |
| **Odp3064** | 5’-CAAATTTCTACTAATGCTTTTATTTTTTC-3’ | 2634-2662  (pNL4-3) | ddPCR US Rv (*pol* RNA) |
| **Odp3106** | 5’-AAGCCAGGAATGGATGGCC-3’ | 2586-2604  (pNL4-3) | ddPCR US probe (*pol* RNA)  6-FAM/MGBNFQ |
| **mRNA levels quantification by qPCR** | | | |
| **Odp3095** | 5’-ACAGTCCATGCCATCACTGCC-3’ |  | Fw qPCR *GAPDH* mRNA |
| **Odp3096** | 5’-GCCTGCTTCACCACCTTCTTG-3’ |  | Rv qPCR *GAPDH* mRNA |
| **Odp3324** | 5’-TGGTGTCAACTGGAAAGCTG-3’ |  | Fw qPCR *DDX1* mRNA |
| **Odp3325** | 5’-CAAAGTGGCAGAGCAAACAA-3’ |  | Rv qPCR *DDX1* mRNA |
| **Odp3326** | 5’-AAGTGACCGCCAATAACGAC-3’ |  | Fw qPCR *FLNA* mRNA |
| **Odp3327** | 5’-GGCGTCACCCTGTGACTTAT-3’ |  | Rv qPCR *FLNA* mRNA |
| **Odp3328** | 5’-CACCGTCTGGATTCTTCCTG-3’ |  | Fw qPCR *HMGB3* mRNA |
| **Odp3329** | 5’-CCCTTTGCACCATCAAACTT-3’ |  | Rv qPCR *HMGB3* mRNA |
| **Odp3330** | 5’-GTGCAGTTTGCTTCACAGGA-3’ |  | Fw qPCR *HNRNPH1* mRNA |
| **Odp3331** | 5’-CCCCAGGTCTGTCATAAGGA-3’ |  | Rv qPCR *HNRNPH1* mRNA |
| **Odp3332** | 5’-GGTGGGGAGAACTACGATGA-3’ |  | Fw qPCR *HNRNPL* mRNA |
| **Odp3333** | 5’-TCAAACTCCACCAGTGCTTG-3’ |  | Rv qPCR *HNRNPL* mRNA |
| **Odp3334** | 5’-AACAGAGGTGGTGGCCATAG-3’ |  | Fw qPCR *HNRNPU* mRNA |
| **Odp3335** | 5’-GTAACTACCACGGCCAGGAA-3’ |  | Rv qPCR *HNRNPU* mRNA |
| **Odp3336** | 5’-ATGAAACTGCGCTCCTGTCT-3’ |  | Fw qPCR *HSP90AA1* mRNA |
| **Odp3337** | 5’-TTCTTCCATGCGTGATGTGT-3’ |  | Rv qPCR *HSP90AA1* mRNA |
| **Odp3338** | 5’-TTACAGCTTCTTCCCTTACA-3’ |  | Fw qPCR *KIF2C* mRNA |
| **Odp3339** | 5’-CCACCTCCATGTCATTCT-3’ |  | Rv qPCR *KIF2C* mRNA |
| **Odp3340** | 5’-AGCCTGAGAAGAAACGGACA-3’ |  | Fw qPCR *KIFC1* mRNA |
| **Odp3341** | 5’-GAACAGCAGGAACTGGCTTC-3’ |  | Rv qPCR *KIFC1* mRNA |
| **Odp3342** | 5’-ATGGCCTACCAGGAATACCC-3’ |  | Fw qPCR *MAP4* mRNA |
| **Odp3343** | 5’-GTATCAGCTGTCGCACTGGA-3’ |  | Rv qPCR *MAP4* mRNA |
| **Odp3346** | 5’-AAGAGATCCCGGAGGTCCTA-3’ |  | Fw qPCR *PLK1* mRNA |
| **Odp3347** | 5’-GCTGCGGTGAATGGATATTT-3’ |  | Rv qPCR *PLK1* mRNA |
| **Odp3348** | 5’-ACGGACCGTTTATCATGAGC-3’ |  | Fw qPCR *PTB* mRNA |
| **Odp3349** | 5’-CATCAGGAGGTTGGTGACCT-3’ |  | Rv qPCR *PTB* mRNA |
| **Odp3354** | 5’-AATCTCAGAGCTTCCCGTCA-3’ |  | Fw qPCR *TOP2A* mRNA |
| **Odp3355** | 5’-TGCCTCTGCCAGTTTTTCTT-3’ |  | Rv qPCR *TOP2A* mRNA |
| **Odp3359** | 5’-GCCAAGCATGTCATCACTCT-3’ |  | Fw qPCR *MCM5* mRNA |
| **Odp3360** | 5’-GCATGATGATGTAGCGGTTC-3’ |  | Rv qPCR *MCM5* mRNA |
| **Odp3361** | 5’-CCTCCAGAACTCCTGTTTAT-3’ |  | Fw qPCR *RBBP7* mRNA |
| **Odp3362** | 5’-GTCGTGACATCTGACTCTT-3’ |  | Rv qPCR *RBBP7* mRNA |
| **Odp3363** | 5’-GACGGAGCTGACCAGACTTT-3’ |  | Fw qPCR *SRP14* mRNA |
| **Odp3364** | 5’-TCTTCCCATCGGTAGCTCTT-3’ |  | Rv qPCR *SRP14* mRNA |
| **Odp3629** | 5’-GTAACCCGTTGAACCCCATT-3’ |  | Fw qPCR *18S* rRNA |
| **Odp3095** | 5’-ACAGTCCATGCCATCACTGCC-3’ |  | Rv qPCR *18S* rRNA |

**Supplemental Table 2:** Primers and probes used in this study. The number, sequence, and usage of each primer are given. Restriction sites are underlined, start and stop codons are in boldfaces.
